# Unraveling the Microenvironment and the Pathogenic Axis of HIF‐1α–Visfatin–Fibrosis in Autoimmune Pancreatitis Using a Single‐Cell Atlas

**DOI:** 10.1002/advs.202412282

**Published:** 2025-01-31

**Authors:** Deyu Zhang, Congjia Ma, Zhen Wang, Yanfang Liu, Zaoqu Liu, Wanshun Li, Yue Liu, Chang Wu, Liqi Sun, Fei Jiang, Hui Jiang, Xiaoju Su, Lisi Peng, Jiayu Li, Xinyue Wang, Hua Yin, Dongling Wan, Yuyan Zhou, Xiaorong Tian, Shiyu Li, Zhendong Jin, Baoan Ji, Zhaoshen Li, Haojie Huang

**Affiliations:** ^1^ Department of Gastroenterology Changhai Hospital Shanghai 200433 China; ^2^ National Key Laboratory of Immunity and Inflammation Naval Medical University Shanghai 200433 China; ^3^ Department of Hepatobiliary Pancreatic Surgery Changhai Hospital Naval Medical University Shanghai 200433 China; ^4^ Department of Pathology Changhai Hospital Naval Medical University Shanghai 200433 China; ^5^ Institute of Basic Medical Sciences Chinese Academy of Medical Sciences and Peking Union Medical College Beijing 100050 China; ^6^ Department of Gastroenterology General Hospital of Ningxia Medical University Ningxia Hui Autonomous Region Yinchuan 750004 China; ^7^ Department of Gastroenterology Sir Run Run Shaw Hospital Zhejiang University School of Medicine Hangzhou 310058 China; ^8^ Department of Cancer Biology Mayo Clinic Jacksonville FL 32224 USA

**Keywords:** autoimmune pancreatitis, HIF1‐α, single‐cell atlas, visfatin

## Abstract

Autoimmune pancreatitis (AIP) is identified as a severe chronic immune‐related disorder in pancreas, including two subtypes. In this study, pancreatic lesions in patients diagnosed as either type 1 AIP or type 2 AIP are examined, and these patients’ peripheral blood at single‐cell level. Furthermore, flow cytometry, immunofluorescence, and functional assays are performed to verify the identified cell subtypes. In type 1 AIP, there is a notable increase in the amount of B cells and plasma cells, and IgG4+ plasma cells are key pathogenic cells of AIP. The differentiation path of naïve‐stage B cells into IgG4+ produced plasma cells is observed, and an increased amount of T helper cells and T follicular helper (Tfh) cells. This study also reveals that HIF‐1α, an activated transcriptional factor, can directly bind to promoter site of NAMPT, promoting higher levels of visfatin production in HIF1A+ classical monocytes. Pancreatic stellate cells can be activated by extracellular visfatin and promote the development of fibrotic response in pancreatic lesions across both AIP subtypes. The current findings shed light on the exploration of dynamic alterations in peripheral blood cells and cell subgroups in pancreatic lesions of AIP, while elucidating a pathogenic cell subset and potential fibrosis mechanism of AIP.

## Graphical abstract

Single‐cell RNA sequencing reveals that type 1 AIP exhibits increased amount and subtypes of B and plasma cells, particularly IgG4+ plasma cells, along with an expansion of Tfh and T helper CD4+ cells. Both AIP types share upregulation of HIF1A+ classical monocytes, which promote pancreatic fibrosis through increased visfatin production.

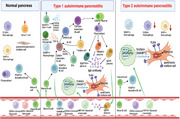

## 1 Introduction

Autoimmune pancreatitis (AIP) is characterized as a special kind of chronic immune‐related disorder of the pancreas that involves damage in the tissue mediated by immune regulation and the development of fibrosis.^[^ ^1^ ^]^ AIP has been categorized into two different subtypes: First one is type 1 AIP, characterized by a dense obliterative phlebitis, lymphoplasmacytic infiltrate, and storiform fibrosis, which is the more common subtype; and type 2 AIP, which histologically shows infiltration of myeloid cell around nearby the area of pancreatic ducts. Notably, storiform fibrosis in pancreatic lesion is a classical pathological feature around the two AIP subtypes.^[^ ^2^ ^]^ Fibrosis in the AIP pancreas appears as an enhancing focal mass lesion that mimics pancreatic cancer, often resulting in misdiagnosis and frequently leading to unnecessary pancreatectomies.^[^ ^3^ ^]^

One of the hallmark features of type 1 AIP is an elevated serum IgG4 concentration, which has been widely recognized as a useful biomarker for the diagnosis and management of this disease. According to a 2016 Japanese survey, 84.5% of patients with type 1 AIP had high serum IgG4 levels.^[^ ^4^ ^]^ However, an elevated serum IgG4 level is not entirely specific to AIP, as it may be elevated in other diseases, including bronchial asthma and atopic dermatitis, and is a feature of ≈10% of cancer cases, including those of pancreatic cancer and cholangiocarcinoma.^[^ ^5^ ^]^ Type 2 AIP lacks the elevated IgG4 serological marker. Its diagnosis often relies on histopathological examination, which typically reveals a granulocytic epithelial lesion (GEL). Therefore, the identification of reliable biomarkers for different types of AIP remains a challenge.^[^ ^1^, ^3^ ^]^

In managing autoimmune pancreatitis (AIP), therapeutic strategies mainly focus on reducing the abnormal immune response and halting the advancement of fibrosis. This is crucial, as unchecked fibrosis can result in permanent organ damage, loss of function, or incorrect diagnoses.^[^ ^6^, ^7^, ^8^ ^]^ The cornerstone of AIP treatment has traditionally been corticosteroids, which exert broad anti‐inflammatory and immunosuppressive effects and have been shown to induce remission in a significant proportion of patients. However, corticosteroid therapy is not without its shortcomings; it can cause considerable side effects, and there is a risk of disease relapse upon tapering or discontinuation of the drugs.^[^ ^6^ ^]^ Additionally, some patients may be steroid resistant or develop steroid‐related complications, highlighting the necessity for the development of alternative targeted treatment options.^[^ ^1^ ^]^

Despite a growing interest in AIP, the pathophysiological mechanisms underpinning this condition remain incompletely understood. The immune microenvironment of AIP plays a crucial role in the progression and maintenance of inflammation and fibrosis. However, the complexity and heterogeneity of the immune cells involved in AIP have not been thoroughly characterized. Recent advances in single‐cell sequencing technologies have provided a new avenue for dissecting the cellular and molecular landscape of AIP at an unprecedented resolution.^[^ ^9^ ^]^ This approach allows for the identification of distinct immune cell populations, their functional states, and the interplay between these populations that may contribute to disease pathogenesis.^[^ ^10^ ^]^ In each subtype of AIP, the immune microenvironment is a dynamic and intricate network in which various immune cells coexist and interact, potentially leading to pathogenic and fibrotic responses. A better understanding of the peripheral blood and pancreatic immune microenvironment is essential for screening novel biomarkers and developing targeted therapies that can modulate the immune response and attenuate fibrosis.

One of the key molecular pathways implicated in AIP‐related fibrosis could be the hypoxia‐inducible factor 1‐alpha (HIF‐1α) pathway. HIF‐1α is a transcription factor that acts in response to hypoxic conditions and regulates the expression of various genes involved in angiogenesis, metabolism, and fibrosis.^[^ ^11^ ^]^ Activation of HIF‐1α can lead to the induction of fibrogenic responses, which are central to the pathology of AIP. VISFATIN, also known as nicotinamide phosphoribosyltransferase (NAMPT), is recognized as an adipocytokine associated with inflammation and fibrosis. Recent studies suggest that VISFATIN may act as a downstream effector of HIF‐1α,^[^ ^12^ ^]^ but the details of this interaction and its impact on fibrosis in AIP patients have yet to be elucidated.^[^ ^13^ ^]^

In this study, we performed single‐cell transcriptome sequencing on 7 type 1 AIP and 2 type 2 AIP patients whose peripheral blood mononuclear cells (PBMCs) and pancreatic tissues were paired and compared with PBMCs from two clinical remission patients and normal pancreatic tissue from healthy donors to determine the complexity of the immune microenvironment in AIP patients. We explored the heterogeneity and alteration of immune cells, their activation states, and the potential involvement of the HIF‐1α and VISFATIN axes in fibrosis. Our findings elucidate the pathogenic mechanisms of AIP and reveal new possibilities for therapeutic interventions targeting the immune system and fibrotic processes.

## 2 Results

### 2.1 A Comprehensive Single‐Cell Transcriptomic Map and Cellular Classifications in Lesions and PBMCs of Type 1 and Type 2 AIP

**Figure 1:**
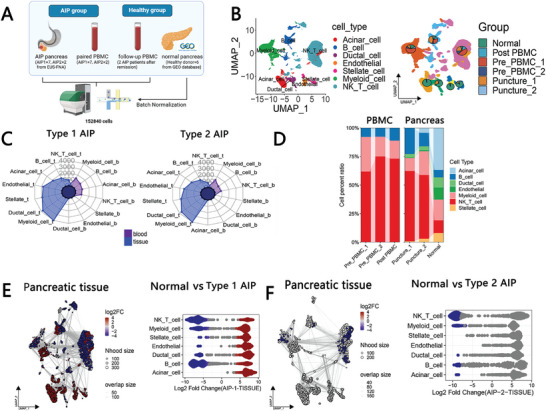
Single‐cell sequencing of pancreatic lesions in type 1 and type 2 autoimmune pancreatitis (AIP) and PBMCs. A) Schematic overview illustrating the study design and main findings. B) UMAP plot depicting all cells from the atlas, with color coding representing distinct cell types (right) and differentiation by group (left). C) Bar graph showing the number of DEGs identified between the AIP group and the normal control group. D) Left: Proportional representation of various differential cell types among PBMCs from patients with type 1 AIP, type 2 AIP, and those in clinical remission after corticosteroid therapy (post‐PBMC). Right: Proportional representation of differential cell types among pancreatic tissues from type 1 AIP, type 2 AIP, and healthy control subjects. E) UMAP plot (right) alongside bee swarm plots (left) illustrating the differences in the abundance of cell types between the normal group and the type 1 AIP group within pancreatic tissues. F) UMAP plot (right) and corresponding bee swarm plots (left) displaying the differences in differential cell type abundances in pancreatic tissues between the normal group and the type 2 AIP group.

The study's design is illustrated in **Figure** **1**A. We obtained PBMCs and corresponding pancreatic lesions from 7 type 1 AIP patients and 2 type 2 AIP patients in active phase. Additionally, we gathered PBMCs from 2 type 1 AIP patients who achieved clinical remission following corticosteroid treatment. The isolated PBMCs and dissociated pancreatic lesions underwent single‐cell transcriptome sequencing (Table S1, Supporting Information), and the resulting data were combined with existing online single‐cell sequencing datasets that included samples from six healthy pancreatic tissues (GSE229413, Figure 1A).^[^ ^14^ ^]^ After conducting multiple quality control steps, we obtained single‐cell data from a total of 152840 cells across all samples (Figure S1A, Supporting Information). These cells were organized into 23 clusters representing seven primary cell types after annotation (Figure 1B; Figure S1B–D, Supporting Information).

Specifically, according to previous studies, JUN, CD3D, CD2, TRAC, CD3G, CD3E, GZMB, CCL5, CZMH, and NKG7 were used as markers to identify NK cell or T cells; S100A9, AIF1, FCER1G, S100A8, FCN1, VCAN, and S100A12 were used as markers to identify myeloid cells; COL13A1, ACTA2, LUM, COL1A1, DCN, and COL1A2 were used as markers to identify pancreatic stellar cells (PSCs); PLVAP and PECAM1 were used as markers to identify endothelial cells; FXYD2, LCN2, EPCAM, KRT19, TSPAN8, and MMP7 were used as markers to identify ductal cells; BANK1, MZB1, IGHA1, MS4A1, and CD79A were used as markers to identify B cells; PRSS1, CELA2A, CPA1, CELA3B and CELA3A were used as markers to identify acinar cells (Figure S1B, Supporting Information). The count of DEGs found between the AIP group and the normal group (post‐PBMC group or normal group, pct >0.35 & |logFC| >0.1) for each cell type is illustrated (Figure 1C; Figure S1E, Supporting Information). We also examined the relationships among the different types of PBMC (Figure S1F, Supporting Information).

Subsequently, we assessed the proportions of various immune cell types present in each type of tissue sample. As depicted in Figure 1D, the percentage of myeloid cells was found to be higher in the pre‐PBMC 1 group compared to the post‐PBMC group. Furthermore, there was an increased proportion of B cells in the pancreatic lesions of patients with type 1 AIP when compared to normal individuals, whereas the pancreatic lesions of type 2 AIP patients exhibited a greater proportion of myeloid cells. To gain deeper insights into the distribution variations among the different immune cell types, we applied the miloR algorithm (Figure 1E,F). Our findings indicated that the abundances of NK cells, T cells, and B cells were significantly elevated in the pancreatic lesions of both type 1 AIP and type 2 AIP patients (Figure 1E). Additionally, we observed a slight increase in the number of myeloid cells specifically in the type 2 AIP patient group (Figure 1F; Figure S1G, Supporting Information). This analysis highlights the distinct immune cell compositions in the pancreatic lesions associated with different types of AIP.

### 2.2 Variations in the Amplification of B‐Cell Subtypes Are Present in the PBMCs and Pancreatic Lesions of Patients with Type 1 AIP

**Figure 2:**
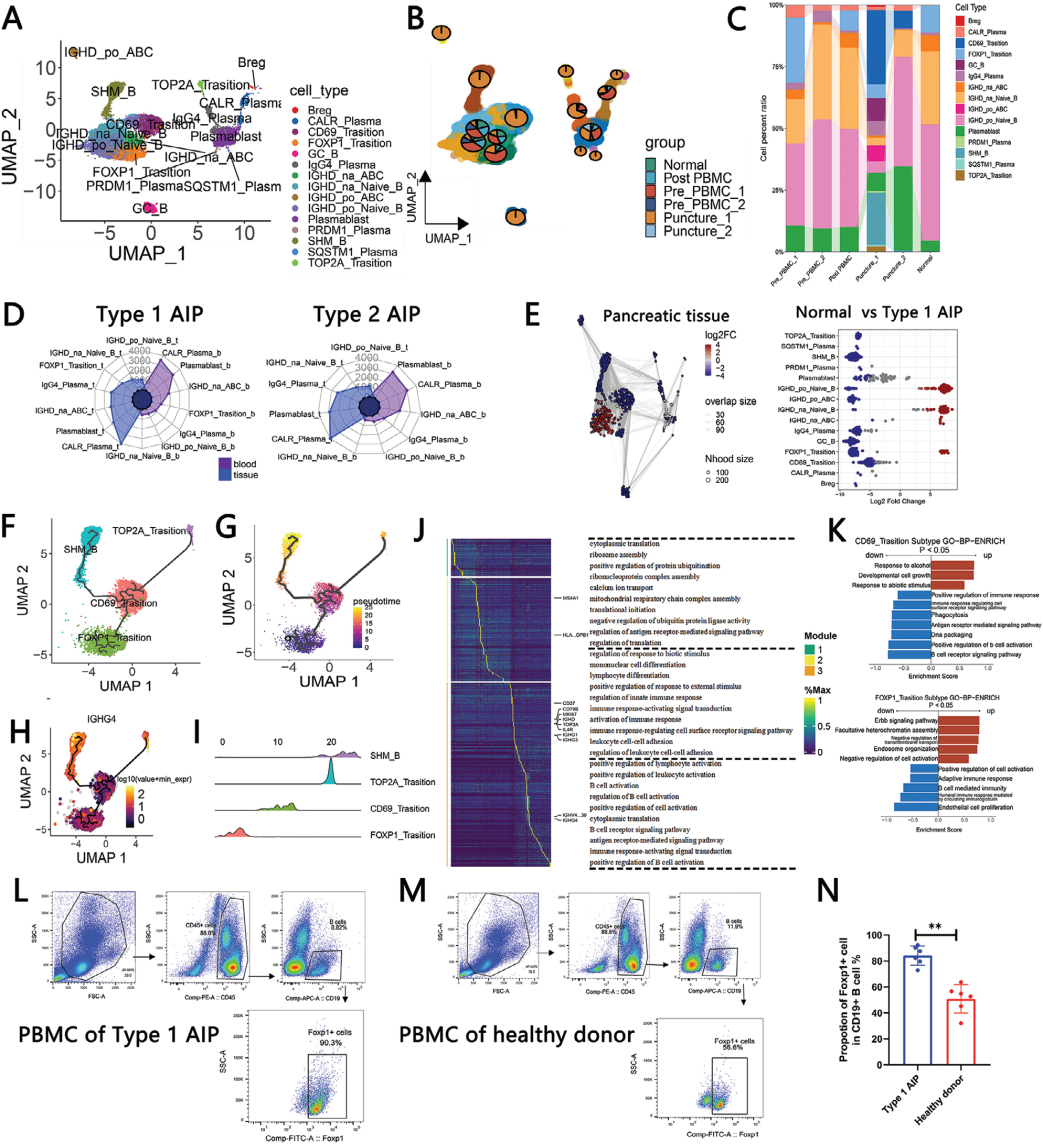
Variability in the amplification of B‐cell subtypes in type 1 autoimmune pancreatitis (AIP). A) UMAP plot illustrating the annotation of various B‐cell subgroups. B) UMAP plot displaying the relative proportions of each B‐cell subgroup within the dataset. C) Bar graph depicting the proportion of each B‐cell subgroup across the different experimental groups. D) Summary of the number of DEGs identified within the B‐cell subgroups when comparing the AIP group to the normal control group. E) UMAP plot (right) accompanied by bee swarm plots (left) showcasing the differences in the abundance of B‐cell subtypes in the pancreatic tissues of the normal group compared to those with type 1 AIP. F) Pseudotime analysis revealing the differentiation trajectory among activated B cells. G) Detailed pseudotime analysis depicting the differentiation trajectory of activated B cells over time. H) Visualization of IGHG4 expression levels in activated B cells. I) Pseudotime analysis results for each individual activated B cell within the dataset. J) Identification of DEGs and functional modules associated with pseudotime analysis, along with enriched GO terms (*p* < 0.01). K) Selected GO terms for the B cells in the type 1 AIP group compared to those in the normal group or post‐PBMC group, as determined by Gene Set Enrichment Analysis (GSEA). L, M) Representative flow cytometry images illustrating the prevalence of FOXP1‐transition B cells in the PBMCs of patients with type 1 AIP compared to healthy donors. N) Histogram demonstrating the proportion of FOXP1‐transition B cells in the PBMCs of type 1 AIP patients versus healthy control individuals. (**p < 0.01).

To explore this, we first identified the various subtypes of B cells present in our current single‐cell atlas. Following our analysis, we distinguished a total of 21 distinct subgroups (Figure S2A, Supporting Information). The expression profiles of specific cell markers associated with these subtypes are depicted in Figure S2B (Supporting Information). Upon annotation, the UMAP plot illustrated the spatial distribution of each annotated cluster (**Figure** **2**A). Specifically, according to previous studies, TGIF1, TGIF2, TGFB1, CD24 and CD38 were used as markers to identify Breg cells; IGHV1‐69D, IGHV4‐39 and IGLV2‐14 were used as markers to identify Somatic hypermutation B‐cells (SHM B‐cells); CD83, CD22 and LRMP were used as markers to identify Germinal Centre B‐cells (GC B‐cells); TOP2A, TUBB, HMGB2, IGHE, ACTB and ACTG1 were used as markers to identify TOP2A transition B‐cells; FOXP1 and IL4R were used as markers to identify FOXP1 transition B‐cells; CD69 and MS4A1 were used as markers to identify CD69 transition B‐cells; SUB1, HSP90B1, CALR and TXN were used as markers to identify CALR plasma cells; PRDM1, IGHG4, IGHG1, IGHG2, IGHG3, and JCHAIN were used as markers to identify PRDM1 plasma cells; SQSTM1, ST6GAL1 and IGHG4 were used as markers to identify SQSTM1 plasma cell; IGHG1, IGHG2, IGHG3 and IGHG4 were used as markers to identify IgG4 plasma cells; IGHA1, JCHAIN, and IGHM were used as markers to identify plasmablasts; IGHD and ITGAX were used as markers to identify IGHD‐positive Age/autoimmunity‐associated B‐cells (IGHD po ABC) and IGHD‐negative Age/autoimmunity‐associated B‐cells (IGHD na ABC); and IGHD and TCL1A were used as markers to identify IGHD‐positive naïve B‐cells (IGHD po naïve B) and IGHD‐negative naïve B‐cells (IGHD na naïve B). Notably, these identified B‐cell subtypes were predominantly found in patients with type 1 AIP (Figure 2B). In particular, we observed a significant increase in the proportion of FOXP1‐positive B cells in the pre‐PBMC 1 group (Figure 2C). Furthermore, the subtypes and proportions of the B‐cell subgroup present in the pancreatic lesions of type 1 AIP patients were the most pronounced compared to other groups (Figure 2C; Figure S2C, Supporting Information). The top 10 marker genes for each identified cluster are shown in Figure S2D (Supporting Information), providing insight into the unique characteristics of these B‐cell subtypes. The expression distributions of various B‐cell marker genes were also visualized (Figure S2E, Supporting Information). Specifically, *IGHG1* is also enriched in IgG4 plasma cells, indicating the potential pathogenicity of IgG4 plasma cells in IgG4 related disease.^[^ ^15^, ^16^ ^]^ We calculated the number of DEGs for each B‐cell subgroup when comparing the AIP group to the normal group (defined as the post‐PBMC group or normal group, with criteria of pct >0.35 & |logFC| >0.1) (Figure 2D; Figure S2F, Supporting Information). Remarkably, nearly all B‐cell subgroups exhibited enrichment within the pancreatic lesions of type 1 AIP patients, underscoring the altered immune landscape in this condition (Figure 2E).

As illustrated in Figure 2F through 2I, the differentiation process of B cells begins with the transition from FOXP1‐positive B cells to CD69‐positive B cells. This initial step is followed by somatic hypermutation (SHM), leading to the emergence of TOP2A transition B cells. Throughout this differentiation process, there is a gradual increase in the expression of the IGHG4 gene, which encodes the pathogenic IgG4 protein associated with type 1 AIP. To further analyze the DEGs involved in this differentiation process, we categorized them into three distinct modules based on their respective differentiation pathways (Figure 2J). GO enrichment analysis revealed that DEGs in module 1 were predominantly associated with processes related to protein translation, indicating a focus on the synthesis of proteins necessary for B‐cell function. In module 2, the DEGs were enriched in pathways related to the differentiation of immune cells, highlighting the role of these genes in guiding B‐cell maturation. Meanwhile, module 3 contained DEGs that were significantly enriched in B‐cell activation processes. Correlation analyses indicated a strong relationship between CD69‐positive B cells and FOXP1‐transition B cells with naïve B cells, suggesting that these subtypes may share developmental pathways or regulatory mechanisms (Figure S2G, Supporting Information). Additionally, we identified GO terms that were significantly enriched in B cells from the type 1 AIP group compared to those found in the normal pancreas or in PBMC samples from patients after remission (with a P‐value threshold of <0.05). Notably, FOXP1‐transition B cells exhibited increased levels of gene expression related to the “negative regulation of cell activation” (Figure 2K), while CD69‐transition B cells showed heightened expression of genes associated with “developmental cell growth” (Figure 2K). As B cells progressed to the SHM stage, the activation of the B‐cell receptor signaling pathway became evident, indicating an important regulatory mechanism during this maturation phase. Furthermore, various proliferation‐related pathways were activated in the context of TOP2A‐transition B cells (Figure S2H, Supporting Information). Collectively, these findings illustrate that during the pathogenesis of type 1 AIP, B‐cell differentiation and maturation occur as a series of gradual and complex processes, characterized by significant changes in various B‐cell subtypes, particularly in the pancreatic lesions.

To further validate the observed upregulation of FOXP1‐transition B cells in the PBMCs of type 1 AIP patients, we collected and analyzed PBMC samples from five individuals diagnosed with type 1 AIP and five healthy donors. The results of our flow cytometry analysis are represented in Figure 2L,M, showcasing a comparison of FOXP1‐transition B cells between the two groups. Our detailed statistical analysis revealed a significant increase in the number of FOXP1‐transition B cells within the PBMCs of patients with type 1 AIP when compared to those from healthy donors (Figure 2N). This finding underscores the relevance of FOXP1‐positive B cells in the context of type 1 AIP, suggesting that these cells may play a crucial role in the disease's immunological landscape. The marked difference in the abundance of these B‐cell subtypes between the two groups further supports our hypothesis regarding the involvement of specific B‐cell populations in the pathogenesis of type 1 AIP.

### 2.3 The Developmental Progression and Subgroup Transformation of Plasma Cells in the Pancreatic Lesions of Patients with Type 1 AIP

**Figure 3:**
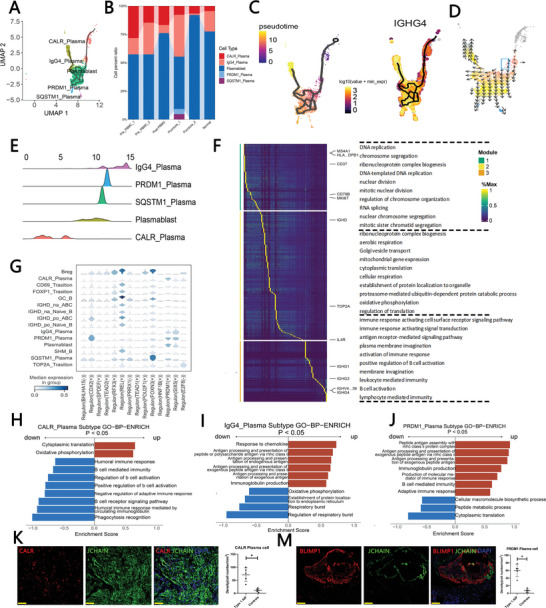
Developmental trajectories and subgroup transitions of plasma cells exhibiting varying differentiation pathways in the pancreatic lesions of patients with type 1 autoimmune pancreatitis (AIP). A) UMAP plot displaying the annotation of distinct plasma cell subgroups identified within the dataset. B) Bar graph illustrating the relative proportions of each plasma cell subgroup present. C) Left: Pseudotime analysis revealing the differentiation trajectory among plasma cells, highlighting the progression of cell states. Right: Visualization of IGHG4 expression levels specifically in plasma cells. D) The differentiation direction of plasma cells, as determined by the Vector algorithm, is shown; the blue box indicates the proposed starting point of the differentiation process. E) Pseudotime analysis results for plasma cells, illustrating their developmental progression over time. F) Identification of DEGs and functional modules through pseudotime analysis, along with enriched GO terms (*p* < 0.01). G) The calculation of putative transcription factors associated with each plasma cell subgroup, derived from analysis utilizing the SENIC algorithm. H–J) Selected GO terms for CALR‐positive plasma cells, IgG4‐positive plasma cells, and PRDM1‐positive plasma cells in the type 1 AIP group compared to those in the normal pancreatic tissue or the post‐PBMC group, as determined by Gene Set Enrichment Analysis (GSEA). K,M) Left: Representative immunofluorescence images depicting CALR‐positive plasma cells and PRDM1‐positive plasma cells within the pancreatic lesions of type 1 AIP patients. Scale bars indicate 50 µm. Right: Quantification of the density of CALR‐positive plasma cells and PRDM1‐positive plasma cells in pancreatic tissues from type 1 AIP patients (n = 5) in comparison to healthy controls (n = 5) (***p < 0.001). Scale bars indicate 50 µm.

Next, we turned our attention to the different subtypes of plasma cells found in the pancreatic lesions associated with type 1 AIP. The distribution of these identified plasma cell subtypes is illustrated in **Figure** **3**A. The accompanying histogram reveals that the proportions of IgG4‐positive plasma cells, PRDM1‐positive plasma cells, and SQSTM1‐positive plasma cells are notably enriched in the pancreatic lesions of type 1 AIP patients (Figure 3B). As depicted in Figure 3C, the differentiation process begins with the transition from CALR‐positive plasma cells to the more activated plasmablast stage. Following this initial conversion, plasmablasts differentiate through three distinct pathways: one leading to the development of PRDM1‐positive plasma cells, another to SQSTM1‐positive plasma cells, and the third to IgG4‐positive plasma cells (Figure 3C,D). The sequence of differentiation for each of these plasma cell subgroups is further outlined in Figure 3E. In our analysis of the DEGs involved in this differentiation process, we categorized the genes into three distinct modules based on their associated differentiation pathways. Module 1 corresponds to the early stages of plasma cell differentiation, while Module 3 represents the terminal stages of differentiation (Figure 3F). The results of GO enrichment analysis indicated that the DEGs within Module 1 are primarily associated with processes related to DNA duplication and nuclear division. Meanwhile, Module 2 contained DEGs enriched in protein translation, and Module 3 featured DEGs linked to B‐cell activation and the activation of immune responses (Figure 3F). Significantly, we observed that the genes PRDM1 and SIX5 were markedly upregulated in the plasmablasts, IgG4‐positive plasma cells, and PRDM1‐positive plasma cells, suggesting their potential role in the pathogenesis of type 1 AIP (Figure 3G; Figure S3A–C, Supporting Information). We identified several significantly enriched GO terms for these plasma cells within the type 1 AIP group, with a statistical significance threshold of *p* < 0.05. Notably, CALR‐positive plasma cells in the type 1 AIP cohort exhibited increased expression of genes related to upregulated “cytoplasmic translation” and downregulated “B‐cell‐mediated immunity” (Figure 3H). Additionally, both IgG4‐positive plasma cells and PRDM1‐positive plasma cells demonstrated upregulated pathways associated with “immunoglobulin production” (Figure 3I,J). Furthermore, SQSTM1‐positive plasma cells indicated a heightened regulation of endocytosis pathways, suggesting an enhanced state of oxidative stress and autophagy within these plasma cells (Figure S3D, Supporting Information). This comprehensive analysis highlights the complex interplay of plasma cell differentiation and function in the context of type 1 AIP, providing valuable insights into the underlying mechanisms of this autoimmune condition.

To further substantiate our findings regarding the presence of CALR‐positive plasma cells and PRDM1‐positive plasma cells, we conducted immunofluorescence analysis on surgical resection specimens obtained from patients diagnosed with type 1 AIP. In this analysis, we employed CALR and JCHAIN as markers to identify CALR‐positive plasma cells, while BLIMP1 (the protein encoded by the PRDM1 gene) and JCHAIN were utilized as markers for PRDM1‐positive plasma cells. The representative images showcasing these cell types are displayed in the left sections of Figure 3K,M. Our quantitative analysis revealed that the proportions of both CALR‐positive plasma cells and PRDM1‐positive plasma cells within the pancreatic tissues of type 1 AIP patients were significantly elevated compared to the corresponding cell populations found in the paracarcinoma tissues, which served as controls. Specifically, the differences were statistically significant, with P‐values of 0.022 for CALR‐positive plasma cells and 0.018 for PRDM1‐positive plasma cells (as indicated in the right section of Figure 3M). These results strongly support our earlier findings and highlight the significant enrichment of these plasma cell subtypes in the pancreatic lesions of type 1 AIP, suggesting their potential role in the disease's immunopathogenesis. This enhanced presence of CALR and PRDM1‐positive plasma cells may reflect the ongoing immune response associated with type 1 AIP.

T follicular helper (Tfh) cells, CD4+ T helper (Th) cells, and a novel inflammatory subtype of T cells associated with NAMPT were discovered in the pancreatic lesions of patients with type 1 AIP.

**Figure 4:**
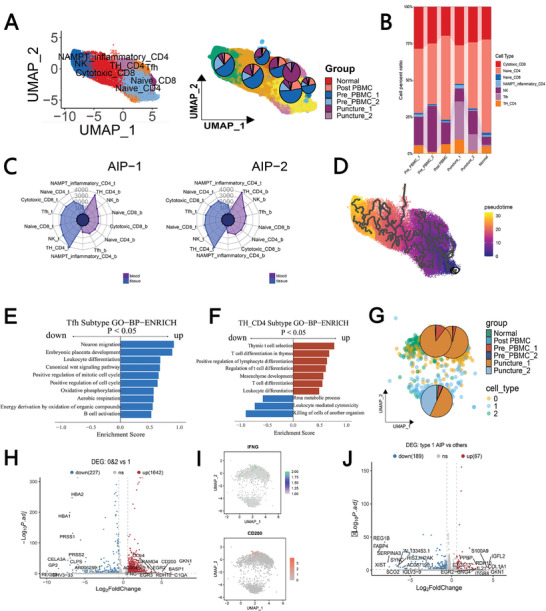
The proliferation of T follicular helper (Tfh) cells, CD4+ T helper (Th) cells, and the identification of a novel inflammatory subtype of T cells associated with NAMPT in pancreatic lesions of type 1 autoimmune pancreatitis (AIP). A) Left: UMAP plot illustrating the annotation of various T‐cell subgroups identified in the analysis. Right: UMAP plot showcasing the relative proportions of each T‐cell subgroup within the dataset. B) Bar graph representing the proportion of each T‐cell subgroup across the different experimental groups examined. C) Summary of the number of DEGs identified when comparing the AIP group to normal samples. D) Pseudotime analysis displayed on a UMAP plot, illustrating the differentiation trajectory of T cells over time. E,F) Selected GO terms for Tfh cells and CD4+ Th cells in the type 1 AIP group as compared to those in normal pancreatic tissue or the post‐PBMC group, determined through Gene Set Enrichment Analysis (GSEA). G) UMAP plot of the Tfh subgroup depicting the proportion of each subgroup within Tfh cells. H) Volcano plot displaying DEGs between clusters 0 & 2 and cluster 1, with the top 10 DEGs highlighted for clarity. I) UMAP plot visualizing the distributions of the genes IFNG and CD200 within Tfh cells, providing insight into their expression patterns. J) Volcano plot illustrating DEGs between type 1 AIP patients and the other experimental groups, with the top 10 DEGs clearly marked for emphasis.

As illustrated in Figure S4A (Supporting Information), we categorized T/NK cells into a total of 20 distinct subgroups. These subgroups were then annotated into seven well‐established T‐cell categories, which include natural killer (NK) cells, cytotoxic CD8+ T cells, follicular helper T (Tfh) cells, CD4+ T helper (Th) cells, NAMPT‐positive inflammatory CD4+ T cells, naïve CD4+ T cells, and naïve CD8+ T cells (Figure S4B, Supporting Information). Our annotation strategy is as follows: according to previous studies, NKG7, KLRD1, and GNLY were used as markers to identify NK cells. CCL5, ZEB2, GZMA, CD8A and GZMH were used as markers to identify cytotoxic CD8+ T‐cells, ICOS, CD69 and CXCR5 were used as markers to identify Tfh cells, and TIGHT, ETS1, STAT1 and GATA3 were used as markers to identify Th CD4+ T‐cells. LTB, LEF1, CD4, CD3E, SELL, IL7R, TCF7, and CD8A were used as markers to identify naïve CD4+ T‐cells and naïve CD8+ T‐cells. Notably, during this process, we identified a novel subtype of CD4+ T cells that is characterized by elevated expression levels of NAMPT and several inflammatory cytokines, specifically S100A6, S100A8, and S100A9 (Figure S4B, Supporting Information). Following the annotation, we generated a UMAP plot that effectively visualizes the distribution of each annotated T‐cell cluster, as shown in **Figure** **4**A. Next, our analysis revealed that the proportions of Tfh cells, CD4+ Th cells, and NAMPT‐positive inflammatory CD4+ T cells were significantly increased in the pancreatic lesions of patients diagnosed with type 1 AIP (Figure 4B; Figure S4C, Supporting Information). To further elucidate the molecular landscape of these T‐cell subgroups, we compiled the top ten DEGs for each annotated T‐cell category, which is presented in Figure S4D (Supporting Information). In examining the DEGs identified across the various T‐cell subgroups, we found that CD4+ Th cells accounted for the largest proportion of DEGs when comparing the AIP group with the normal control group (comprising either the post‐PBMC group or other normal group samples, with criteria set for pct > 0.35 & |logFC| > 0.1). This is illustrated in Figure 4C and Figure S4E,F (Supporting Information). The predominance of DEGs within the CD4+ Th cells highlights their potential significance in the immune response observed in type 1 AIP, suggesting that these cells may play a critical role in the pathophysiology of the disease. Recent studies show the importance of CXCR3+ Th1 cells and CD4+ CTLs in IgG4‐related disease,^[^ ^17^ ^]^ we next verified the expression of these two cell subtype in our single‐cell AIP data. We observed the genes and gene marker enrichment scores of Th1(CXCR3, CD4)^[^ ^18^ ^]^ and CD4‐CTL cells(IL7R, KLRG1, PRF1, TNF, NCR3, GZMA, GZMK)^[^ ^19^ ^]^ respectively, and it could be observed that the proportion of Th1 in the whole Th cells was ≈25%, and no typical CD4CTL cells were observed (Figure S4G left and middle, Supporting Information). In our investigation, we also conducted pseudotime analysis to elucidate the differentiation trajectory of T cells, as shown in Figures S4G right, S4H,I (Supporting Information). The expression profiles of several key markers for each T‐cell subgroup are illustrated in Figure S4H,I, (Supporting Information) providing insight into the unique characteristics of these cells during the differentiation process. We identified significantly enriched GO terms for certain T‐cell subgroups in the type 1 AIP cohort compared to those found in normal pancreatic tissues or in PBMCs after remission, with a significance threshold set at *p* < 0.05. Among the noteworthy findings, the Tfh cells from the type 1 AIP group exhibited enrichment in pathways related to “Leukocyte differentiation” (Figure 4E). Additionally, the CD4+ Th cells within the type 1 AIP group demonstrated an upregulation of pathways associated with “T‐cell differentiation” (Figure 4F). The transcription factors pertinent to each T‐cell subtype are detailed in Figure S4J,K (Supporting Information).

We then shifted our focus to the cytokine factors secreted by Tfh cells and CD4+ Th cells. The data pertaining to Tfh cells are presented in Figure 4G, which illustrates the distribution of these cells across various clusters, highlighting that the proportion of Tfh cells associated with type 1 AIP was notably highest in clusters 0 and 2 (Figure 4G). To gain a deeper understanding of the molecular profiles within these clusters, we computed the top ten upregulated genes in clusters 0 and 2 in comparison to those in cluster 1. Among these top upregulated cytokine‐related genes, we identified CD200 and IFNG, both of which displayed significant increases (Figure 4H,I; log fold change >1.5, adjusted *p* value <0.05). Furthermore, we analyzed the DEGs in CD4+ Th cells between the type 1 AIP group and the other study groups, with IL21 being prominently detected among the top upregulated genes (Figure 4J; log fold change >1.5, adjusted *p* value < 0.05). Additionally, we validated the presence of NAMPT‐positive inflammatory CD4+ T cells within the pancreatic tissues of both type 1 AIP patients and the control group, further corroborating the significance of these cell populations in the context of the disease (Figure S4L, Supporting Information).

### 2.4 HIF‐1α‐Positive Classic Monocytes Are Abundant in the Pancreatic Tissues of Patients with Both Type 1 and Type 2 AIP and Exhibit Proinflammatory Characteristics

**Figure 5:**
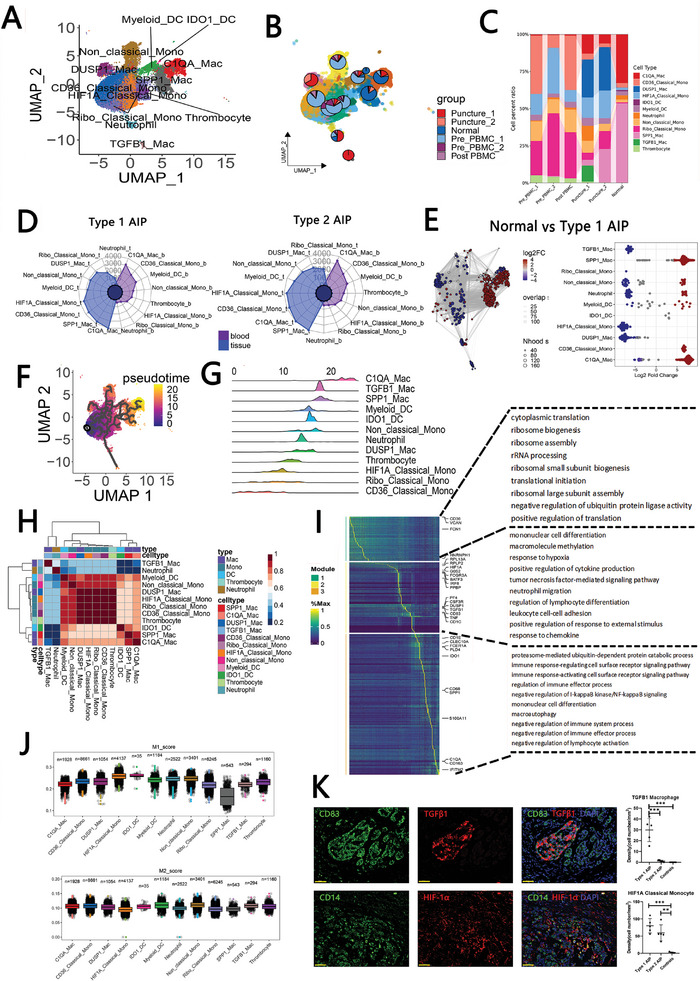
Enrichment of classic HIF‐1α‐positive monocytes in patients with type 1 and type 2 autoimmune pancreatitis (AIP), exhibiting a proinflammatory phenotype. A) UMAP plot illustrating the annotation of various myeloid cell subgroups identified within the dataset. B) UMAP plot showcasing the relative proportions of each myeloid cell subgroup, providing insight into their distribution. C) Bar graph representing the proportion of each myeloid cell subgroup across the different experimental groups, including type 1 AIP, type 2 AIP, post‐PBMC, and normal groups. D) Summary of the number of DEGs identified between the AIP group and normal group, encompassing both type 1 and type 2 AIP, and comparing them to the post‐PBMC group or the normal group. E) UMAP plot (left) alongside bee swarm plots (right) illustrating differential abundances of cell types in the pancreatic tissues of the normal group versus the type 1 AIP group. F) Pseudotime analysis revealing the differentiation trajectory among myeloid cells, indicating their developmental progression over time. G) Pseudotime analysis results for each individual myeloid cell subgroup, highlighting their unique differentiation paths. H) Heatmap representing the correlation analysis between different myeloid cell subgroups, showcasing their interrelationships. I) Identification of DEGs and functional modules derived from pseudotime analysis, along with enriched GO terms (*p* < 0.01), to elucidate biological processes involved. J) Evaluation of signature scores for M1 and M2 polarization in each myeloid cell subgroup, reflecting their inflammatory states. K) Right: Representative immunofluorescence image depicting NAMPT‐positive inflammatory CD4+ T cells present in the pancreatic lesions of type 1 AIP patients. Scale bars indicate 50 µm. Left: Quantification of the density of TGFB1‐positive macrophages and classic HIF1A‐positive monocytes in the pancreatic tissues of type 1 AIP patients (n = 5), type 2 AIP patients (n = 5), and healthy controls (n = 5), with statistical significance indicated (**p < 0.01, ***p < 0.001).

Then, we proceeded to identify various subgroups of myeloid cells, for which the top ten genes characterizing each cluster are displayed in Figure S5A,B,G (Supporting Information). According to previous studies, IFITM2 and CSF3R were used as markers to identify neutrophils. PF4 and PPBP were used as markers to identify thrombocytes. IFR8, BATF3, and IDO1 were used as markers to identify IDO1‐positive DCs. PLD4, FCER1A, CLEC10A, CD1E, and CD1C were used as markers to identify myeloid DCs. TNF, CD83, and TGFB1 were used as markers to identify TGFB1‐positive macrophages. DUSP1 was used as a marker to identify DUSP1‐positive macrophages. CD163 and C1QA were used as markers to identify C1QA‐positive macrophages. S100A11, SPP1, and CD68 were used as markers to identify SPP1‐positive macrophages. FCGR3A was used as a marker to identify nonclassic monocytes. GOS2 and HIF1A were used as markers to identify classic HIF1A‐positive monocytes. RPLP2, RPL13A, and HNRNPH1 were used as markers to identify classic Ribo‐positive monocytes. FCN1, VCAN, and CD36 were used as markers to identify classic CD36+ monocytes. Through our analysis, we successfully classified these groups into 12 recognized myeloid cell subgroups, as illustrated in **Figures** **5**A and S5C,D (Supporting Information). Our findings revealed that C1QA‐positive macrophages and SPP1‐positive macrophages constituted the largest proportions of immune cells within the normal pancreatic tissue samples (Figure 5B,C). However, in patients diagnosed with type 1 AIP, we noted an increase in the proportion of TGFB1‐positive macrophages. In both type 1 and type 2 AIP patients, there was a significant rise in the proportion of HIF1A‐positive classic monocytes, while the proportions of C1QA‐positive macrophages and SPP1‐positive macrophages were found to be decreased (Figure 5B,C). When analyzing the DEGs across the myeloid cell subgroups, we observed that the overall number of DEGs in pancreatic tissues was greater than that in other groups (Figure 5D). Notably, the C1QA‐positive macrophages, SPP1‐positive macrophages, CD36‐positive classic monocytes, and HIF1A‐positive classic monocytes exhibited the highest numbers of DEGs among all the myeloid cell subgroups analyzed (Figures S5E, Supporting Information). Previous research has demonstrated that plasmacytoid dendritic cells (pDCs) that produce IFN‐alpha and IL‐33 play a proinflammatory and pathogenic role in Autoimmune Pancreatitis (AIP) and IgG4‐related diseases.^[^ ^20^ ^]^ Additionally, M2 macrophages expressing IL‐33 have been implicated in the pathogenesis of IgG4‐related disease.^[^ ^21^, ^22^ ^]^ We next would like to identify whether pDCs or M2 macrophages are activated. The enrichment scores of pDCs gene makers (“CLEC4C”, “TCF4”,“IFNA1”,“IL33”)^[^ ^23^ ^]^ and M2 macrophage gene markers(“CD163”,“TLR7”)^[^ ^21^ ^]^ was calculated by Seurat function AddModuleScore, respectively. It can be observed that ≈80% of C1AQ macrophages are M2 macrophages, but no CLEC4C+TCF4 double‐positive pDCs mentioned in the citation were found, and no myeloid cells were found to transcribe IL33 (Figure S5F, Supporting Information). Additionally, we found that the proportions of TGFB1‐positive macrophages, nonclassic monocytes, neutrophils, HIF1A‐positive classic monocytes, and DUSP1‐positive macrophages were decreased in normal pancreatic tissues but significantly elevated in the pancreatic lesions of type 1 AIP patients (Figure 5E). Furthermore, HIF1A‐positive classic monocytes and DUSP1‐positive macrophages also showed increased proportions in the pancreatic lesions of type 2 AIP patients (Figure S5H, Supporting Information). Interestingly, HIF1A‐positive monocytes emerged as the only myeloid cell subtype that was consistently upregulated in PBMCs and pancreatic lesions of both type 1 and type 2 AIP patients (Figure S5I, Supporting Information). The potential transcription factors associated with each myeloid cell subgroup are depicted in Figure 5J. Figure 5F,G illustrates a proposed differentiation pathway, beginning with the transformation of classic CD36‐positive monocytes and classic Ribo‐positive monocytes into classic HIF1A‐positive monocytes. These classic monocytes further differentiate into DUSP1‐positive macrophages, which can eventually mature into SPP1‐positive, TGFB1‐positive, and C1QA‐positive macrophages. We also conducted a relevant analysis which revealed two distinct expression models during the differentiation process (Figure 5H). The DEGs identified along these pathways were categorized into three modules based on their differentiation trajectories. Enriched GO analysis for these modules indicated that DEGs in module 1 were associated with protein translation, while DEGs in module 2 were found to be enriched in monocyte differentiation, as well as responses to hypoxia and chemokines (Figure 5I). These findings were supported by lower M1 scores observed in C1QA‐positive macrophages and SPP1‐positive macrophages (Figure 5J). To further validate our findings, we performed immunofluorescence to confirm the presence of TGFB1‐positive macrophages and HIF1A‐positive classic monocytes in the pancreatic tissues of type 1 AIP, type 2 AIP, and control patients. A representative image is displayed on the left side of Figure 5K. Notably, the proportion of TGFB1‐positive macrophages in the pancreatic tissues of type 1 AIP patients was significantly higher compared to that of type 2 AIP patients (*p* = 0.00056, right part of Figure 5K) and paracarcinoma tissue (controls, *p* = 0.00016, right part of Figure 5K). Furthermore, the proportion of TGFB1‐positive macrophages in type 1 AIP patients was significantly greater than that of HIF1A‐positive classic monocytes in paracarcinoma tissue (type 1 AIP patients and controls, *p* = 0.0023, right part of Figure 5K). Similarly, in type 2 AIP patients, the proportion of TGFB1‐positive macrophages was also significantly greater than that of HIF1A‐positive classic monocytes in paracarcinoma tissue (type 2 AIP patients and controls, *p* = 0.00014, right part of Figure 5K).

The activation of visfatin in macrophages induced by HIF‐1α is a shared mechanism that contributes to fibrosis, occurring through interactions between macrophages and pancreatic stellate cells in both type 1 and type 2 AIP.

**Figure 6:**
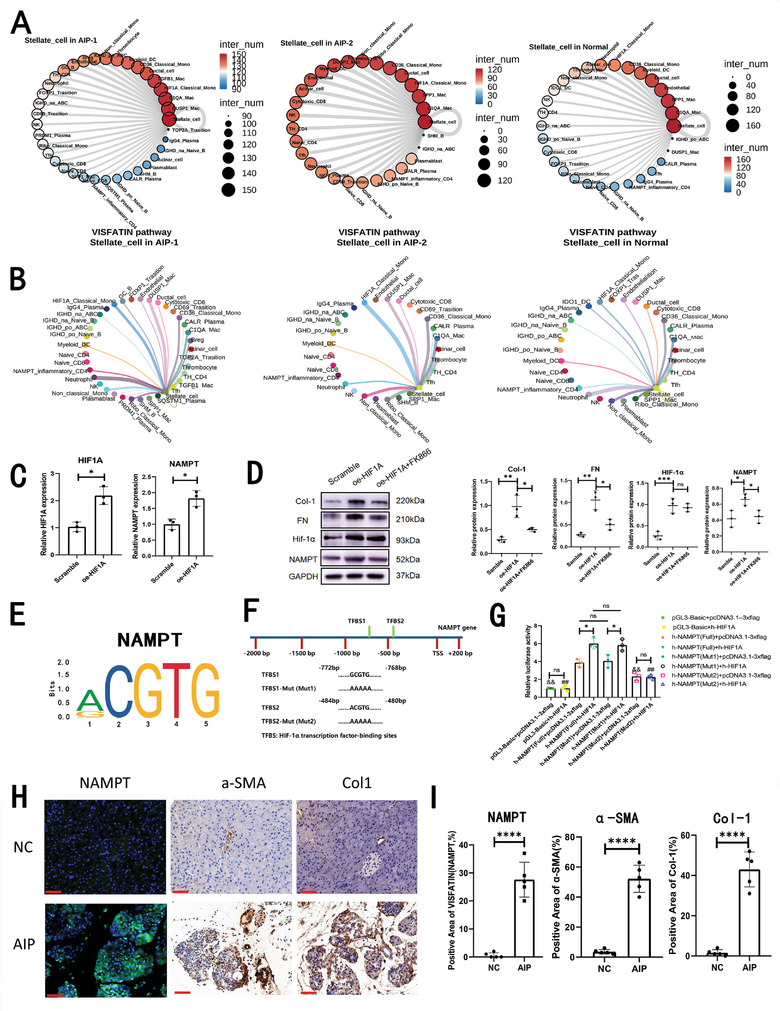
HIF‐1α‐induced activation of visfatin in macrophages contributes to fibrosis through interactions between macrophages and pancreatic stellate cells in both type 1 and type 2 autoimmune pancreatitis (AIP) patients. A) A summary of the number of interaction pairs between various cell subgroups and pancreatic stellate cells is presented for patients with type 1 AIP (left), type 2 AIP (middle), and normal control groups (right). B) The interaction strength of the visfatin signaling pathway between different cell subgroups and pancreatic stellate cells is depicted for type 1 AIP (left), type 2 AIP (middle), and normal groups (right). C) Relative mRNA expression levels of HIF1A and NAMPT are compared in RAW264.7 cells between scramble and HIF1A‐overexpressing groups. D) Analysis of protein expression levels of collagen‐1 (Col‐1), fibronectin (FN), HIF‐1α, NAMPT, and GAPDH in primary MPSCs after co‐culture with various RAW264.7 groups, including the scramble group, HIF‐1α overexpression group (OE‐HIF1A), and the HIF‐1α overexpression group with exogenous FK866 (OE‐HIF1A+FK866). Statistical significance is indicated as ns: not significant (*p* > 0.05); *: *p* < 0.05; **: *p* < 0.01. E) Visualization of enriched motif binding to HIF‐1α on the NAMPT promoter region, highlighting potential regulatory elements. F) Potential binding sites of HIF‐1α (TFBS1 and TFBS2) on the NAMPT promoter were predicted using the JASPAR database, including sequences for both full‐length and mutated promoter constructs (mut1, mut2). G) Results of dual‐luciferase assays measuring the luciferase activity of NAMPT promoter constructs (full, Mut1, and Mut2). Data are expressed as means ± SDs (n = 3), with statistical significance denoted as ns: not significant (*p* > 0.05); *: *p* < 0.05; **: *p* < 0.01, & *p* < 0.05 when compared to the h‐NAMPT (full) + pcDNA3.1–3xflag group; ^##^ *p* < 0.01 when compared to the h‐NAMPT (full) + h‐HIF1A group. H) Representative immunofluorescence and immunohistochemical images displaying the expression of NAMPT, α‐smooth muscle actin (α‐SMA), and collagen‐1 (Col‐1) in untreated control mice (NC) versus AIP mice (AIP). Scale bars indicate 50 µm. I) Quantification of the proportions of NAMPT‐, α‐SMA‐, and Col‐1‐positive areas in untreated mice (NC) compared to AIP‐treated mice (AIP), with n = 5 for each group. Statistical significance is indicated with **** denoting *p* < 0.0001.

Next, we investigated the status of cell–cell interactions among different cell types within the pancreatic microenvironment. Our analysis indicated that the number of inferred interactions within the type 2 AIP and type 1 AIP groups was significantly higher than that observed in the normal group, as demonstrated in Figure S6A (Supporting Information). The strength of incoming and outgoing interactions is visually represented in Figure S6B (Supporting Information). Previous research has indicated a role for injured acinar cells in the progression of autoimmune pancreatitis (AIP),^[^ ^24^ ^]^ and our current findings further reveal that IgG4‐positive plasma cells are the primary producers of IgG4 with potential pathogenic effects in type 1 AIP. Consequently, we focused on the interactions between other cell types in AIP and these two specific cell types (Figure S6C,D, Supporting Information). Our results showed increased interactions between IgG4‐positive plasma cells and pancreatic stellate cells in the type 1 AIP group compared to those in either the type 2 AIP group or the normal group (Figure S6C, Supporting Information). Additionally, we found that the number of interactions between acinar cells and macrophages was greater in both the type 1 and type 2 AIP subgroups compared to the normal group (Figure S6D, Supporting Information). A comprehensive overview of the interaction pairs among various cell types in normal samples, type 1 AIP samples, and type 2 AIP samples is provided in Figure S6E (Supporting Information). Subsequently, we predicted the interaction pairs between each identified immune cell type and pancreatic stellate cells. The results indicated that several myeloid cell subgroups, including C1QA‐positive macrophages, SPP1‐positive macrophages, and HIF1A‐positive classic monocytes, exhibited a higher number of interaction pairs compared to other cell types (**Figure** **6**A). Notably, the number of interaction pairs between HIF1A‐positive classic monocytes and pancreatic stellate cells was found to be significantly elevated in the AIP groups (both type 1 AIP and type 2 AIP) compared to the normal group. Furthermore, prior studies have established that visfatin serves as the extracellular form of NAMPT, demonstrating interaction capabilities with various cell types and playing critical roles in biological processes. Our analysis revealed a robust interaction strength in the visfatin (NAMPT) pathway between pancreatic stellate cells and classic HIF1A‐positive monocytes (Figure 6B, indicated by the red frame; Figure S6E,F, Supporting Information).

We then aimed to elucidate the mechanism underlying visfatin production and its effects on the activation of pancreatic stellate cells. To this end, we created a mouse macrophage RAW264.7 cell line that stably overexpresses HIF1A. We found that the mRNA expression levels of both HIF1A and NAMPT were significantly upregulated in the HIF1A‐overexpressing cell line (Figure 6C). Subsequently, we examined how these HIF1A‐overexpressing macrophages influence the fibrotic phenotype of pancreatic stellate cells through a coculture system. Western blot analyses demonstrated that the overexpression of HIF1A in macrophages could lead to the upregulation of NAMPT, as well as fibrosis‐related proteins such as collagen‐1 (Col‐1) and fibronectin (FN) in MPSCs. Notably, the elevated expression of these fibrosis‐related proteins in MPSCs was reversed upon the addition of the NAMPT inhibitor FK866 (Figure 6D; Figure S7A, Supporting Information). Given that HIF‐1α is a crucial transcription factor that can induce NAMPT transcription, we hypothesized that HIF‐1α might directly bind to the NAMPT promoter to initiate its transcription. To explore this, we utilized the JASPAR database to predict potential binding sites for HIF‐1α on the NAMPT promoter. Our analysis identified one motif of NAMPT that was overwhelmingly enriched in the datasets (Figure 6E), and two HIF‐1α transcription factor‐binding sites (TFBS1 and TFBS2) in the NAMPT promoter displayed high scores (Figure 6F). We subsequently cloned the full‐length NAMPT and corresponding NAMPT mutants into the pGL3‐Basic vector. Comparatively, the group overexpressing h‐HIF1α showed a marked increase in the expression of HIF1α in HEK‐293T cells, both at the mRNA and protein levels (Figure S7B, Supporting Information), indicating that h‐HIF1α effectively upregulates HIF‐1α expression in these cells. Importantly, compared to the control group (pGL3‐Basic+pcDNA3.1–3xFlag), luciferase activity was significantly elevated in the h‐NAMPT (Full)+pcDNA3.1–3xFlag group and the h‐NAMPT (Full)+h‐HIF1α group. The luciferase activity in the h‐NAMPT (Full)+h‐HIF‐1α group showed an even greater increase compared to the h‐NAMPT (Full)+pcDNA3.1–3xFlag group. These results suggest that HIF‐1α can bind directly to the NAMPT promoter to facilitate NAMPT transcription.

We created mutants for the two HIF‐1α binding sites on the human NAMPT promoter. The results showed that the luciferase activity of the h‐NAMPT (Mut2)+pcDNA3.1–3xFlag group was decreased compared to that of the h‐NAMPT (Full)+pcDNA3.1–3xFlag group, while the luciferase activity for h‐NAMPT (Mut1)+pcDNA3.1–3xFlag remained unchanged. Similarly, the luciferase activity of the h‐NAMPT (Mut2)+h‐HIF‐1α group also decreased compared to that of the h‐NAMPT (Full)+h‐HIF‐1α group, while the luciferase activity for h‐NAMPT (Mut1)+h‐HIF‐1α did not change. These findings indicate that HIF‐1α specifically binds to TFBS2 in the NAMPT promoter, thereby regulating NAMPT transcription.

To validate these findings in vivo, we established an AIP mouse model (Figure S7C, Supporting Information). We utilized immunofluorescence and immunohistochemistry techniques to assess the expression of NAMPT and fibrosis‐related proteins, including alpha‐smooth muscle actin (α‐SMA) and collagen‐1, in both the AIP mice and normal control mice (n = 5). A representative image is presented in Figure 6H, showing the expression of NAMPT (visfatin), α‐SMA, and collagen‐1. Quantitative analysis revealed that the areas positively expressing visfatin, α‐SMA, and collagen‐1 were significantly greater in the pancreas of AIP mice compared to normal controls (Figure 6I).

### 2.5 Visfatin Facilitated Fibrosis of PSCs by Activating the TLR4 Receptor and the ROS‐Dependent TGF‐β Signaling Pathway in AIP

**Figure 7:**
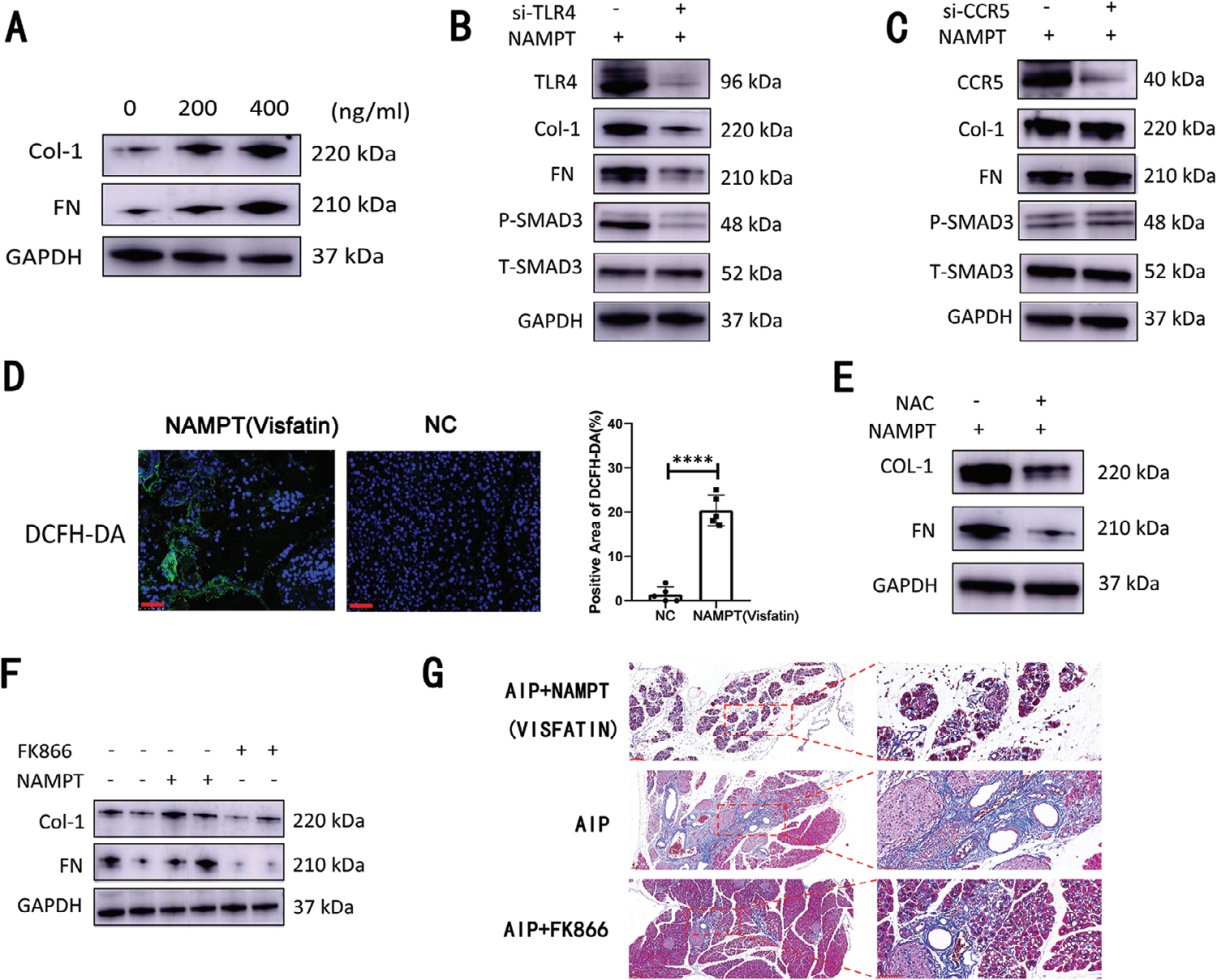
Visfatin enhances fibrosis in pancreatic stellate cells via activation of the TLR4 receptor and the ROS‐induced TGF‐β signaling pathway in autoimmune pancreatitis (AIP). A) Analysis of protein expression levels for collagen‐1 (Col‐1), fibronectin (FN), and GAPDH following stimulation with exogenous NAMPT, demonstrating the fibrogenic response. B) Protein expression levels of collagen‐1 (Col‐1), fibronectin (FN), phosphorylated SMAD3 (p‐SMAD3), total SMAD3 (T‐SMAD3), and GAPDH in human pancreatic stellate cells (HPSCs) from both the scramble control group and the TLR4‐knockdown group (si‐TLR4) after treatment with exogenous NAMPT. C) Similar analysis of protein expression levels for collagen‐1 (Col‐1), fibronectin (FN), phosphorylated SMAD3 (p‐SMAD3), total SMAD3 (T‐SMAD3), and GAPDH in HPSCs from the scramble control group and the CCR5 knockdown group (si‐CCR5) following stimulation with exogenous NAMPT. D) Left: Representative images showing DCFH‐DA and DAPI staining in the pancreas of AIP mice compared to normal control mice. Right: Quantitative comparison of DCFH‐DA‐positive areas in the pancreas between AIP mice and normal controls (n = 5), with scale bars indicating 50 µm. Statistical significance is denoted by ****, indicating *p* < 0.0001. E) Assessment of protein expression levels for collagen‐1 (Col‐1), fibronectin (FN), and GAPDH in HPSCs from both the scramble control and TLR4‐knockdown groups after treatment with exogenous NAMPT. F) Evaluation of protein expression levels of collagen‐1 (Col‐1), fibronectin (FN), and GAPDH in cells treated with or without exogenous NAMPT or the NAD+ synthesis inhibitor FK866. G) Representative images of Masson staining showcasing the pancreatic architecture in AIP mice treated with or without exogenous NAMPT or FK866. Scale bars are shown as left: 100 µm, right: 50 µm.

**Figure 8:**
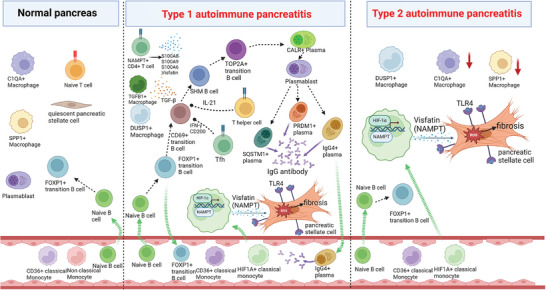
Schematic representation illustrating the mechanisms and all identified cell subtypes involved in the development of AIP.

We proceeded to investigate the mechanisms underlying visfatin‐induced fibrosis in PSCs. Our experiments demonstrated that visfatin significantly upregulated the expression of fibrosis‐related proteins, including collagen‐1 and fibronectin (FN), while promoting fibrotic changes in a dose‐dependent manner, as illustrated in **Figures** **7**A and S7D (Supporting Information). Building on prior research indicating that visfatin interacts with two notable receptors—TLR4 and CCR5—we utilized siRNA technology to knock down the expression of these receptors in human pancreatic stellate cell (HPSC) lines. Following the exogenous addition of NAMPT (visfatin), we observed a marked activation of the TGF‐β signaling pathway alongside evident fibrotic changes in the HPSCs. Notably, the knockdown of TLR4 expression, as opposed to CCR5, partially inhibited the activation of the TGF‐β pathway and the associated fibrotic response (Figure 7B,C; Figure S7E,F, Supporting Information). Previous studies have identified the critical role of reactive oxygen species (ROS) in TLR4‐mediated fibrotic processes, and our analysis confirmed that ROS levels within the pancreas of AIP mice were significantly elevated compared to those in normal controls (Figure 7 and n = 5). To further verify the contribution of ROS to visfatin‐induced fibrosis at the cellular level, we conducted additional experiments. The introduction of exogenous NAMPT led to the induction of a fibrotic phenotype in HPSCs, which could be partially reversed by administering the ROS antagonist N‐acetylcysteine (Figure 7E; Figure S7G, Supporting Information). Subsequently, we examined the effects of exogenous NAMPT (visfatin) and FK866, a NAMPT inhibitor, on the fibrotic phenotypes in AIP mouse models. Our findings revealed that the administration of exogenous NAMPT exacerbated the fibrotic features associated with AIP, while treatment with FK866 alleviated these fibrotic manifestations (Figure 7F; Figure S7H, Supporting Information; n = 3). A representative image displaying these results is shown in Figure 7G. Then, we assessed the levels of visfatin in the plasma of untreated patients with type 1 AIP and type 2 AIP, as well as healthy donors serving as controls.∖ These findings highlight the potential role of visfatin in the pathogenesis of AIP. The new cell subsets identified, along with the underlying mechanisms elucidated throughout this study, are summarized in **Figure** **8**.

## 3 Discussion

To the best of our previous knowledge, our research is the first study to provide a comprehensive single‐cell atlas of AIP. Additionally, it is the first to utilize single‐cell sequencing technology on human pancreatic tissue obtained via endoscopic ultrasound‐guided fine needle aspiration (EUS‐FNA). Previous research has indicated the application of bulk sequencing on pancreatic samples DNA or RNA samples acquired from benign tissues and malignant lesions.^[^ ^25^, ^26^ ^]^ However, those studies primarily focused on bulk sequencing methods, whereas our research offers insights into an optimal EUS‐FNA‐based diagnostic approach by dissecting pancreatic tissues at the single‐cell level.

In line with prior studies,^[^ ^2^, ^4^, ^27^ ^]^ our findings underscore the critical role of B‐cell activation and differentiation in type 1 AIP, with minimal B‐cell activation observed in type 2 AIP. Our in‐depth examination of B‐cell and plasma cell subtypes in type 1 AIP has uncovered unique immunological profiles that could contribute to the disease's pathogenesis. Specifically, we noted a rise in FOXP1+ transitional B cells in both the peripheral blood and pancreatic lesions of patients with type 1 AIP. Prior studies have highlighted the crucial role of the transcription factor Foxp1 in the early stages of B‐cell development.^[^ ^28^ ^]^ Our results suggest that the elevated amount of pancreatic FOXP1+ transition B cells represent initial stage in pathogenic differentiation occurring in both the pancreatic lesions and peripheral blood of patients with type 1 AIP. The distinct cellular profiles in premature B cells identified, particularly the increased presence of FOXP1+ transitional B cells suggest potential biomarkers that could enhance diagnostic accuracy for type 1 AIP. In view that previous study has identified relying on serum IgG4 concentrations for diagnosing type 1 AIP can result in misdiagnosis rates exceeding 10% and omission rates of 15.5%,^[^ ^29^ ^]^ further studies need to be done and to evaluate the diagnostic significance of FOXP1+ transition B cell levels in AIP patients’ peripheral blood.

Our results indicate a marked differentiation path in alteration of B cells arising from type 1 AIP patients’ pancreatic lesion. This path initiates from naïve B cells progressing followed by the formation of CD69+ transition B cells after alteration into FOXP1+ transition B cells. Prior research has demonstrated that CD69 play a pivotal role in B‐cell activation.^[^ ^30^, ^31^ ^]^ Subsequently, CD69+ transition B cells undergo somatic hypermutation (SHM) and alter into a transition subtype with high expression of TOP2A and several other proliferation markers; previous studies have highlighted their proliferative capabilities.^[^ ^32^ ^]^ Furthermore, our investigation supports the notion that this cell type serves as the unique precursor for plasma cells.

An intriguing discovery concerning, in type 1 AIP patients, a kind of mature plasma cells display a phase marked by an increased expression of proteins which play a role in protein production, and locate in endoplasmic reticulums, such as CALR, while not showing a similar rise in immunoglobulin levels. This particular subtype of plasma cells has not been previously reported in patients with type 1 autoimmune pancreatitis (AIP) and may serve as a promising target for strategies aimed at reducing the release of pathogenic immunoglobulin G4 (IgG4) from mature plasma cells. Our research has categorized three distinct types of pathogenic plasma cells responsible for IgG4 production: PRDM1+ plasma cells, SQSTM1+ plasma cells, and IgG4+ plasma cells. All of these cell types derive from plasmablasts and exhibit a significant capacity for IgG4 synthesis. Notably, the predominant subtype, identified as IgG4+ plasma cells, has the ability to migrate into the peripheral bloodstream of individuals suffering from type 1 AIP. This migration could provide an explanation for the observed increases in serum IgG4 levels, as well as the presence of extrapancreatic manifestations associated with this condition. We have included the distribution of IgG1 among B cell subtypes (Figure S2E, Supporting Information) and observed that IgG4 plasma cells also show the highest expression of IgG1. Given that both IgG1 and IgG4 are pathogenic antibodies associated with AIP, and that IgG1 demonstrates stronger pathogenicity,^[^ ^15^ ^]^ these findings underscore the significance of IgG4 plasma cells in the development of AIP. By providing the first comprehensive single‐cell atlas of AIP, we reveal critical insights into B‐cell activation and differentiation that align with existing literature highlighting the role of B‐cells in various autoimmune conditions. For instance, similar findings in other autoimmune diseases, such as rheumatoid arthritis (RA) and systemic lupus erythematosus (SLE),^[^ ^33^, ^34^ ^]^ have demonstrated the importance of B‐cell subsets in disease progression, suggesting a broader paradigm where B‐cell dysregulation underlies multiple autoimmune pathologies.

Additionally, our current research reported the proliferation and expansion of T follicular helper and CD4+ T helper cells occur in type 1 AIP patients’ pancreas, and the phenomenon was not observed in the pancreatic lesion of type 2 AIP patients. Previous research has also emphasized the essential role of Tfh cells in type 1 AIP.^[^ ^35^ ^]^ Another study indicated that type 1 AIP exhibits a predominant T helper cell immune response compared to type 2 AIP.^[^ ^36^ ^]^ Tfh cells are crucial for priming B cells to initiate extrafollicular and germinal center antibody responses, which are fundamental for affinity maturation and the maintenance of humoral memory.^[^ ^37^ ^]^ The specifics of T helper cell involvement in B‐cell activation remain to be fully elucidated, and these mechanisms should be clarified in type 1 AIP patients. Furthermore, we identified a novel CD4+ T‐cell subgroup with high expression levels of NAMPT and some inflammatory cellular cytokines, which is enriched in type 1 AIP patients’ pancreatic lesions. The expansion of this CD4+ T‐cell population suggests a complex network of cellular interactions driving the inflammatory response in type 1 AIP patients. These findings indicate that therapeutic strategies targeting T‐cell phenotypes may represent an alternative approach for treating AIP. Our identification of enriched T follicular helper (Tfh) and CD4+ T helper cells in type 1 AIP parallels findings from studies on other autoimmune disorders, including RA,^[^ ^38^ ^]^ SLE^[^ ^39^ ^]^ and Multiple Sclerosis,^[^ ^40^, ^41^ ^]^ that emphasize the role of these cells in orchestrating immune responses. Moreover, building on recent studies highlighting the importance of CXCR3+ Th1 cells and CD4+ CTLs in IgG4‐related disease,^[^ ^17^ ^]^ we verified the expression of these cell subtypes in our single‐cell AIP dataset and found that Th1 cells constituted ≈25% of the total Th cell population, while typical CD4+ CTLs were not observed (Figure S4G left and middle, Supporting Information).

We also observed an increase in TGFB1+ macrophages in type 1 AIP patients’ pancreatic lesion. Some previous studies suggested that TGF‐β is predominantly secreted by fibroblasts, rather than macrophages.^[^ ^42^, ^43^, ^44^ ^]^ Thus, the presence of TGFB1+ macrophages in type 1 AIP patients’ pancreas is noteworthy. According to recent findings by Tao et al., macrophage‐to‐myofibroblast transition may occur in certain pathological processes.^[^ ^45^ ^]^ We hypothesize that TGFB1+ macrophages represent a transitional cell type involved in this process within pancreatic lesions of type 1 AIP patients. However, further experimentation is necessary to confirm this hypothesis.

Previous research has demonstrated that pDCs that produce IFN‐alpha and IL‐33 play a proinflammatory and pathogenic role in Autoimmune Pancreatitis (AIP) and IgG4‐related diseases.^[^ ^20^ ^]^ Additionally, M2 macrophages expressing IL‐33 have been implicated in the pathogenesis of IgG4‐related disease.^[^ ^21^, ^22^ ^]^ In our research, we aimed to assess the activation of pDCs and M2 macrophages, and found that while ≈80% of C1AQ macrophages were identified as M2 macrophages, we did not observe any CLEC4C+TCF4 double‐positive pDCs or myeloid cells expressing IL‐33, suggesting a potential absence of these proinflammatory cell types in AIP.

Crucially, our analysis revealed an enrichment of HIF1A+ classic monocytes in both the peripheral blood and pancreatic lesions of types 1 and 2 AIP. Markers such as CD36(+), VCAN(+), and FCGR3A(‐) indicate high expression of monocyte markers without the expression of macrophage‐related markers. This suggests that this monocyte subgroup is capable of chemotactic migration from the peripheral blood into the pancreas in both types of AIP. The expansion of HIF1A+ classic monocytes underscores shared pathogenic mechanisms between the two AIP subtypes, highlighting that the underlying mechanisms of type 1 and type 2 AIP cannot be easily segregated. HIF1α, encoded by HIF1A, is a key transcription factor that is upregulated in response to hypoxic stress.^[^ ^46^ ^]^ An intriguing question arises regarding the factors that induce the formation of HIF1A+ classical monocytes. Previous research have indicated that intestinal dysbiosis may contribute to the development of AIP by activating pDCs,^[^ ^47^ ^]^ and the disruption of the intestinal barrier further worsens AIP by facilitating the translocation of Staphylococcus sciuri into the pancreas.^[^ ^48^ ^]^ Another previous study indicated that the expression of HIF1α is induced in LPS‐activated macrophages, playing a significant role in the expression of proinflammatory genes.^[^ ^49^ ^]^ Combining these studies, we hypothesize that the expression of HIF1α in macrophages during AIP may be attributed to the translocation of gut microbiota to the pancreas, which in turn activates the infiltration of monocytes by bacterial LPS. However, this hypothesis requires further validation to confirm its accuracy. To date, the role of HIF1α in AIP has not been investigated. Interestingly, a recent study related to chronic pancreatitis reported that hypoxia and HIF‐1α activation in the pancreas are crucial for the activation of pancreatic stellate cells and the development of pancreatic fibrosis.^[^ ^50^ ^]^ Through assessments of cell‐cell interactions, we also established that HIF1A+ classic monocytes are vital effector cells interacting closely with pancreatic stellate cells, with visfatin playing a significant role in this process across both types of AIP. Visfatin, first identified by Fukuhara et al. in 2005 as an insulin‐mimicking adipokine in mice, was later found to be synonymous with pre‐B‐cell colony‐enhancing factor.^[^ ^51^, ^52^ ^]^ Additionally, visfatin exhibits nicotinamide phosphoribosyltransferase (Nampt) activity, which is essential for converting nicotinamide to nicotinamide mononucleotide (NMN) and subsequently to NAD+.^[^ ^53^ ^]^ There are two forms of Nampt: iNampt (the intracellular form), which is important for NAD‐dependent enzyme activities, and visfatin (the extracellular form), which influences interactions between organs. Recent studies have detected visfatin in various tissues and cells, including those of the immune system,^[^ ^54^ ^]^ chondrocytes,^[^ ^55^ ^]^ and amniotic epithelial cells.^[^ ^56^ ^]^ Importantly, visfatin has been shown to promote fibrosis in multiple organs, including the liver and heart.^[^ ^57^, ^58^ ^]^ Additionally, visfatin has been found to be highly expressed in various autoimmune diseases, including one study in RA^[^ ^59^ ^]^ and another study in autoimmune thyroiditis.^[^ ^60^ ^]^ However, the serum levels and potential roles of visfatin in AIP remain to be fully elucidated. In our current study, we demonstrated that HIF‐1α directly activates NAMPT (the gene encoding visfatin) by binding to its promoter and that secreted visfatin can stimulate the fibrosis of pancreatic stellate cells through TLR4 activation and ROS generation. Administration of exogenous visfatin worsened pancreatic fibrosis in an AIP mouse model, and this adverse effect could be mitigated by treatment with a visfatin inhibitor. By developing visfatin inhibitors, we may offer new strategies for managing relapsing or refractory cases of AIP, potentially reducing the progression of fibrosis and improving patient outcomes. These insights underscore additional studies are necessary to explore the clinical and translational applications of visfatin inhibitors in reducing pancreatic fibrosis in type 1 and type 2 AIP patients, to pave the way for more effective diagnostic and therapeutic approaches.

Our study has several limitations. First, the sample size of patients diagnosed with type 2 AIP for single‐cell sequencing was relatively small. Second, neutrophils, which are important cell types in type 2 AIP, were not adequately captured in the single‐cell sequencing data from type 2 AIP patients in this study. Third, as a single‐cell study based on pancreatic lesion acquired by endoscopic ultrasound‐guided fine‐needle aspiration, this approach inevitably leads to loss of information of pancreatic parenchymal cells, different from the single‐cell study of pancreatitis in mouse pancreas.^[^ ^61^ ^]^ Future research should enroll a larger cohort of both type 1 and type 2 AIP patients to address these limitations.

In summary, our research has illuminated the complete developmental pathway from B cells to disease‐causing plasma cells in type 1 autoimmune pancreatitis (AIP). Moreover, we observed a notable increase in T follicular helper (Tfh) cells and CD4+ T helper cells in individuals diagnosed with type 1 AIP. A key finding of our study is the demonstration that HIF‐1α has the ability to directly interact with the promoter region of the NAMPT gene, which enhances the production and elevated expression of extracellular visfatin in HIF1A+ classic monocytes. This mechanism plays a significant role in the fibrosis of pancreatic stellate cells, highlighting a shared fibrotic pathway in pancreatic lesions associated with both type 1 and type 2 AIP. These insights present a promising therapeutic target for future treatment strategies of AIP.

## 4 Experimental Section

### Patients and Samples

Between October 2022 and October 2023, PBMCs and paired pancreatic lesions from seven active type 1 AIP patients and two active type 2 AIP patients without glucocorticoid therapy were collected during EUS‐FNA procedures performed at Changhai Hospital in Shanghai, China. The MR and CT imagine these patients are diffusely or locally enlarged pancreas, autoimmune pancreatitis was highly suspected. According to the guideline,^[^ ^62^ ^]^ EUS‐FNA was exerted to collect the biopsy specimen of pancreatic lesion in these patients. Additionally, PBMCs from 2 type 1 AIP patients with clinical remission after corticosteroid therapy were also collected. These 2 type 1 AIP patients are enduring symptoms including obstructive jaundice, abdominal pain, back pain, and initial oral prednisolone dose for induction of remission is 0.6 mg k^−1^g/day, which is administered for 4 weeks and then gradually tapered as the guideline suggested.^[^ ^62^ ^]^ No other treatment was performed on these 2 type 1 AIP patients previously. Comprehensive details for each sample are systematically cataloged in Table S1 (Supporting Information). Between October 2013 and October 2023, five normal pancreatic tissues from patients with intraductal papillary mucinous neoplasm(para‐tumor tissue) were harvested during pancreatectomy surgery and preserved in paraffin block. Five pancreatic tissues from patients with type 1 AIP and five pancreatic tissues from patients with type 2 AIP were also harvested during Whipple surgery, and they were preserved in paraffin block. These patients sought medical attention due to abdominal pain, jaundice, and other symptoms. Imaging examinations found a solid mass and serum CA199 is above normal, highly suggested pancreatic malignancy, but EUS‐FNA examinations of the pancreatic lesions did not find malignant cells and suggested possibility of AIP. Based on the previous consensus statement and the possibility of AIP combining with pancreatic cancer, these patients were suggested and they agreed to endure Whipple surgery (Table S2, Supporting Information).^[^ ^63^, ^64^ ^]^ These pancreatic tissues were used to perform immunofluorescence.

To perform flow cytometry on PBMC and confirm the serum level of Visfatin, PBMC was also collected from initial treatment AIP patients and five healthy donors (Table S3, Supporting Information). Type 1 or type 2 AIP diagnoses were confirmed based on the pathological result of EUS‐FNA or surgical tissue and previous diagnostic consensus of AIP.^[^ ^65^ ^]^ Prior to participating in the study, all patients were required to provide written informed consent, ensuring that they understood the nature of the research and agreed to take part. The ethical aspects of this research were carefully reviewed and approved by the Ethics Committee of Changhai Hospital, which issued approval number CHEC‐2022‐239. For a thorough overview of each sample involved in the study, comprehensive details and relevant information have been systematically organized and can be found in Table S1 (Supporting Information). Additionally, six healthy pancreatic tissues were downloaded from GEO database as normal pancreatic tissue (GSE229413).^[^ ^14^ ^]^

### Isolation of PBMCs

Peripheral blood (5 mL) was collected from participants into EDTA‐containing tubes to prevent coagulation. The collected blood was then diluted with an equal volume of 1× c (PBS). This diluted blood was carefully layered over an equal volume of Ficoll in a 50 mL centrifuge tube. The sample was centrifuged at 2000 rpm for 20 min at 20 °C using a horizontal rotor centrifuge with the brake set to “off”. Following centrifugation, the interface layer containing PBMCs was carefully aspirated and transferred to a new 15 mL centrifuge tube. The PBMCs were washed by adding 10 mL of 1× PBS and centrifuging at 300 g for 10 min. The supernatant was discarded, and the washing step was repeated twice with 5 mL of 1× PBS each time, followed by centrifugation at 300 g for 10 min. After discarding the final wash supernatant, the cells were resuspended in 1 mL of RPMI 1640 medium supplemented with 0.04% bovine serum albumin (BSA) (10‐040‐CVR, Corning, USA). Cell concentration and viability were assessed using a Luna Automated Cell Counter (Logos Biosystems, USA) and trypan blue exclusion staining method.

### Tissue Processing and Cell Isolation

Tissues were washed twice with precooled RPMI 1640 medium supplemented with 0.04% BSA under sterile conditions. The tissue was placed in freshly prepared enzymatic digestion solution and incubated at 37 °C in a humidified incubator for 30 to 60 min, referencing relevant literature for enzymatic digestion times. The suspension was mixed by inverting every 5 to 10 min during digestion. After digestion, the cell suspension was filtered through a 40 µm cell strainer (BD Biosciences, USA) once or twice, followed by centrifugation at 300 g for 5 min at 4 °C. The pellet was resuspended in an appropriate volume of growth medium and mixed with an equal volume of red blood cell lysis buffer (130‐094‐183, MACS, USA). The mixture was allowed to stand at 4 °C for 10 min, then centrifuged at 300 g for 5 min, and the supernatant was discarded.

### Cell Preparation and Single Cell Sequencing

ScRNA‐seq libraries were constructed and sequenced with MobiNova‐100 (MobiDrop, Zhejiang, China) platform according to the manufacturer's instructions by OE Biotech Co., Ltd. (Shanghai, China). Raw sequencing files were processed using the MobiVision software pipeline to obtain gene expression matrices by demultiplexing cellular barcodes, mapping reads to the genome and transcriptome using the STAR aligner, and down‐sampling the reads as required to generate normalized aggregate data across samples.

### scRNA‐Seq Data Processing, Cluster Annotation and Data Integration

Filtered count matrices were converted to sparse matrices using the Seurat package (v4.4.0),^[^ ^66^ ^]^ and cells expressing less than 500 genes as well as more than 5 000 genes, cells’ proportion of mitochondria is higher than 10%, the cells’ total number of mRNA molecules detected less than 500, the cells’ erythrocyte content higher than 3% and genes expressing less than three cells, were excluded from the downstream analysis. The “SoupX” method from the SoupX package (v1.6.2)^[^ ^67^ ^]^ and the “doubletFinder_v3” method from the DoubletFinder package (v2.0.3)^[^ ^68^ ^]^ were applied for additional cell filtering. Filtered data were then log normalized and scaled, with cell–cell variation due to UMI counts and percent mitochondrial reads regressed out.

To avoid batch effects between six healthy pancreatic tissues downloaded from GSE229413 and our local sequencing data, fast, sensitive, and accurate integration of single‐cell data was made with Harmony package (v1.0.3).^[^ ^69^ ^]^ Harmony employs an iterative clustering approach to align cells from different batches. Initially, it combines the batches and projects the data into a reduced‐dimensional space via PCA. The algorithm then iteratively removes dataset‐specific effects through four main steps:

1. It groups cells into clusters using a variant of soft k‐means clustering for efficient cell clustering.
2. It calculates a global centroid for each cluster and a dataset‐specific centroid.
3. Using these centroids, it determines a correction factor for each dataset.
4. This correction factor is then applied to adjust each cell's data.

This process repeats until convergence. In an analysis, the examples from the Harmony package in R were followed and integrated Harmony into the Seurat workflow, using the maximum of 50 clusters and 100 iterations. The top 20 normalized Harmony vectors in PCA space were then utilized for further assessments.

After this, a total of 2 000 features for anchoring (the “FindVariableFeatures” function) and 40 dimensions for alignment (“Integrate Data”) were used. Cell clustering was performed by “FindClusters” function at a resolution of 0.1. Dimensionality reduction was performed with “Run UMAP” function and visualized by Uniform Manifold Approximation and Projection (UMAP). For subgroup cell clustering, cells of different types were extracted separately and clustered by their respective first 30 principal components (PCs) using different resolutions based on visual inspection.

### Identification of Signature Genes

The “FindAllMarkers” function in Seurat was applied to identify specific genes for each cell subset. For the selection of marker genes specific to each cell cluster/subset, the log2 fold change (log2FC) between two groups (a cell cluster/subset versus other cells) was calculated using the “FindMarkers” function with the Wilcoxon rank‐sum test (default parameters). Then, gene markers of cell types and each cell subgroup were chosen according to previous studies and the cell subgroup was annotated according to these gene markers.

### Pathway Analysis

Differentially expressed genes (DEGs) were detected by the “FindAllMarkers” function in Seurat. GSEA was conducted using the fGSEA package(v1.24.0),^[^ ^70^ ^]^ gene pathway analysis was performed according to the Gene Ontology (GO) iteration system with data table and fGSEA. For calculation of gene sets enrichment score, the marker enrichment score was calculated using the Seurat function AddModuleScore.

### Pseudotime Analysis by Monocle3

B cell, T cell, and Myeloid cell developmental trajectories were inferred using Monocle3 (version 1.0.0) with default parameters as recommended by the developers.^[^ ^71^ ^]^ First, integrated gene expression matrices from specific cell type were exported from Seurat into Monocle to construct a CellDataSet. Second, the “setOrderingFilter” function was applied to sort cells with the variable genes identified by the function of “differentialGeneTest” (cutoff of q < 0.01). Finally, after dimensionality reduction using the “reduceDimension” function (using the “DDRTree” reduction method), a series of representative key role genes were revealed along the differentiation progress by the “Heatmap” function. Dimensionality reduction was performed with no normalization and the “DDRTree” reduction method in the “reduceDimension” step.

### Cell–Cell Interaction Analysis

Cell–cell interactions among the cell types were estimated by CellChat (v1.6.1)with default parameters.^[^ ^72^ ^]^ Changes in signaling pathways between type 1 AIP, type 2 AIP, and normal group, especially those involving Acinar cell, Stellate cell, and IgG4 plasma were highlighted.

### Differential Abundance Testing

Differential abundance tests were made by miloR(v1.6.0)^[^ ^73^ ^]^ with default parameters. K‐nearest neighbor graphs were made by “buildGraph” function(k = 30, d = 30). Neighborhoods on a graph were defined by “makeNhoods” function(k = 30, d = 30, prop = 0.2).

### Differential Genes Calculation

“FindMarkers” function (logfc.threshold = 0, min.pct = 0.35) was used to calculate the differential genes between normal and pathological samples. Radar and scatter plots were used to visualize the number of differential genes in each cell subgroup.

### Analysis of Gene Expression Correlation among Subgroups

Spearman correlation coefficient was used to measure the correlation of gene expression among subgroups, and pheatmap (v1.0.12) was used to visualize the correlation among subgroups.

### Single‐Cell Regulatory Network Speculation

To infer the regulatory activity of TF in the subpopulation, the pySCENIC software package (release 0.12.1) was used for gene regulatory network analysis.^[^ ^74^ ^]^ Regulatory module regions were identified by inferring co‐expression between TF and genes containing TF‐binding motifs in their promoters. The co‐expression modules of transcription factors and the provided expression matrix genes were inferred based on grnboost2. Subsequently, TF‐motif enrichment analysis was performed to identify the target of transcription factor (TF), and its corresponding direct action was obtained. Finally, AUCell was used to score each regulon activity for each cell.

### Immunofluorescence and Immunohistochemical Staining (IHC)

For IHC and Immunofluorescence (IF), multiple antibodies are used in these experiments, including anti‐CD83(ab244204, Abcam, Cambridge, UK), anti‐TGF‐β1(ab315254, Abcam, Cambridge, UK), anti‐CD14(ab183322, Abcam, Cambridge, UK), anti‐HIF‐1α(ab308433, Abcam, Cambridge, UK), anti‐NAMPT(ab236874, Abcam, Cambridge, UK), anti‐α‐SMA(ab7817, Abcam, Cambridge, UK), anti‐Collagen1(ab316222, Abcam, Cambridge, UK), anti‐BLIMP1 (ab241568, Abcam, Cambridge, UK), anti‐JCHAIN(ab269855, abcam company, USA). For IHC, paraffin‐embedded pancreatic tissue sections were dewaxed and rehydrated twice in phosphate‐buffered saline (PBS) for 15 min. The sections were incubated with 0.3% H2O2 for 15 min to block endogenous peroxidases, washed twice in PBS, incubated with 30 g L⁻^1^ BSA for 10 min to prevent nonspecific binding of antibodies, and then incubated with antibodies. Finally, diaminobenzidine (DAB) was added as a chromogen, followed by haematoxylin. For immunofluorescence (IF), the antibodies were used to detect the cell markers. Then, the slides of the paraffin block were incubated with multiple antibodies and counterstained with 4′,6‐diamidino‐2‐phenylindole (DAPI) (ab228549, Abcam, Cambridge, UK). Finally, the slides were observed under a fluorescence microscope.

### Cell Culture

Human pancreatic stellate cells (HPSCs) were generously provided by Prof. Logsdon CD from the Anderson Cancer Center's Department of Cancer Biology, Houston, Texas, USA.^[^ ^75^ ^]^ Mouse pancreatic stellate cells (MPSC) were acquired as per the previous report.^[^ ^76^ ^]^ In summary, pancreatic tissues from mice were collected through a sterile procedure and then cleansed using fetal bovine serum (FBS, SH30070, Gibco, USA). These tissues were subsequently sectioned into blocks of 0.5–1 mm^3^ and placed in 6‐well plates for cultivation. The medium in which they were grown was refreshed on day two. Primary pancreatic stellate cells (PSCs) emerged from the blocks, which were then transferred to fresh 6‐well plates for continued cultivation. Only MPSCs from passages 2 to 5 were utilized in the experiments. Raw 264.7 cells were sourced from the American Type Culture Collection (ATCC, VA, USA). Human Embryonic Kidney (HEK‐293) cells were also sourced from the American Type Culture Collection (ATCC, VA, USA). Both HPSCs, MPSCs, HEK‐293 T cells, and Raw 264.7 cells were cultured in DMEM supplemented with 10% FBS and 1% penicillin‐streptomycin, in a 5% CO₂ environment at 37 °C, with medium changes every other day. Exogenous NAMPT was purchased from MedChemExpress LLC, USA (No. HY‐P70353).

### Cell Transfection

Small interfering RNA (siRNA)‐to knock down TLR4 (si‐TLR4), CCR5 siRNA (si‐CCR5) and negative control NC (si‐NC) were designed and synthesized by RiboBio (Guangzhou, China). HPSCs were transfected with the siRNAs using Lipofectamine 2000 (Thermo Fisher, USA). Cells were seeded in a complete medium ≈12 h before transfection. Then, siRNAs mixed with Lipofectamine 2000 were added to the cells with fresh Opti‐MEM medium (gibco, USA). SiRNAs were transfected at a concentration of 50 nM. After 6 h, the medium containing siRNAs and Lipofectamine 2000 was replaced with a complete medium. As for transfection of overexpression plasmid, HIF1A‐expression construct (NM_0 01530) and pENTER empty vector construct were designed and provided by Genechem (Shanghai, China). MPSCs were placed into each well of 6‐well plate and were cultured overnight. Then, the cells were transfected with the plasmid using Lipo3000 and P3000 (Thermo Fisher, USA) and cultured for 36 h before protein extraction. Expression was detected via using western blot.

### Macophage and Pancreatic Stellar Cells Co‐Culture Assay

In a 6‐well Transwell system, MPSCs (1 × 10^6^) were plated on the lower chamber, whereas different groups of RAW264.7 cells (1 × 10^6^) were added to the upper chamber; the pore size was 0.4 µm. All cells were grown in DMEM supplemented with 10% FBS, 50 U mL^−1^ penicillin (Gibco, USA), and 50 µg  mL^−1^ streptomycin (Gibco, USA). After 6 h co‐culture, FK866 (HY‐50876, MedChemExpress LLC, USA) was added to one group of co‐culture systems. Then, each group of the MPSCs was harvested from the lower chamber after 48 h.

### Real‐Time Quantitative PCR

HEK‐293 T and RAW264.7 cells were suspended in Trizol reagent (Invitrogen), and the total RNA was extracted according to the manufacturer's instructions. Reverse transcription was performed using a Reverse Transcription Kit (Toyobo, Osaka, Japan). A SYBRGreen RT‐PCR Kit from Toyobo was used for quantitative real‐time quantitative PCR analysis with the Step One PLUS Real‐time PCR system (Applied Biosystems, USA), according to the manufacturer's instructions. Gene‐specific primers were used to detect human HIF1A (forward primer: 5‐CTACTAGTGCCACATCATCACCA‐3; reverse primer: 5‐GATTGCCCCAGCAGTCTACAT‐3), mouse HIF1A (forward primer: 5‐CAAGAAACCACCCATGAC‐3; reverse primer: 5‐GGCTCATAACCCATCAAC‐3) and mouse NAMPT (forward primer: 5‐GCAGAAGCCGAGTTCAACATC‐3; reverse primer: 5‐TTTTCACGGCATTCAAAGTAGGA‐3). The samples were normalized against endogenous human GAPDH (forward primer: 5‐TGCCCCCATGTTCGTCA‐3; reverse primer: 5‐CTTGGCCAGGGGTGCTAA‐3), mouse GAPDH (forward primer: 5‐CCTTCATTGACCTCAACTAC‐3; reverse primer: 5‐CCAAAGTTGTCATGGATG‐3), and fold changes were calculated using the formula 2^−△Ct^.

### Dual‐Luciferase Reporter Assay

HEK‐293 T cells were suspended and seeded at the appropriate density (1.0 × 10^4^) in 96‐well plates. The binding sites of HIF‐1α on the NAMPT promoter were predicted in JASPAR database (JASPAR, http://jaspar.genereg.net/). The relative profile threshold is set as 90%. Two binding sites were identified. Then, when the number of cells reached 70%, Dual‐Luciferase reporter assay was performed to explore the binding relationship between HIF‐1α and *NAMPT* promoter. The *NAMPT* promoter region fragments (Full, Mut1 and Mut2) were constructed into the pGL3‐basic vector, and *HIF‐1A* was subcloned into the pcDNA3.1–3x flag vector (Chudian Bio, China). The firefly luciferase plasmids h‐HIF‐1α (Full, Mut1, Mut2) or pGL3‐Basic (NC) were co‐transfected with HIF1αeukaryotic expression vectors (containing h‐HIF1α or pc‐DNA3.1–3x flag) and the pRL‐TK renilla luciferase vector (internal reference) into HEK‐293 T cells. After 48 h, cell lysates were extracted, and firefly and Renilla luciferase activities were also detected using Dual‐Luciferase Reporter Assay Kits (DE‐WLRA‐122, Chudian Bio, China). The luminescence signal was detected using a multifunctional microplate reader (SuPerMax3100, Shanghai Flash Spectrum Biological Technology Co., Ltd, China). The experiment was repeated three times.

### Western Blot

RIPA buffers were used to extract total protein from cells or pancreatic tissue, and BCA protein assay kit was used to determine total protein concentration. The same mass of protein (24 µg) is added to the sample tank and then subjected to SDS‐polyacrylamide gel electrophoresis. All the protein was transferred to polyvinylidene difluoride membranes and blocked with 3% BSA for 1 h at room temperature. They are then incubated with an anti‐HIF‐1α(catalog # 340462, ZENBIO, China), NAMPT(catalog # DF6059, Affinity, China), FN(catalog # sc‐8422, Santa Cruz, USA), Collagen‐1(catalog # 72026, Cell Signaling Technology, USA), CCR5(catalog # AF6339, Affinity, China), TLR4(catalog # AF7017, Affinity, China), P‐SMAD3(catalog # r380775, ZENBIO, China), T‐SMAD3(catalog # r25743, ZENBIO, China). The gray value of the detected bands was quantified by using ImageJ software. The experiment was repeated three times.

### Intracellular ROS Assay

Cell climbing sheets were prepared, and the cells were subsequently cultured in a treated medium. Afterward, the old medium was discarded and replaced with fresh medium infused with 0.1% 2′, 7′‐dichlorofluorescin diacetate (DCFH‐DA). These sheets were then incubated at 37 °C, shielded from light, for 20 min. To finalize the process, the cells adhering to the sheets were washed three times with PBS, and images were captured using a fluorescence microscope.

### Animals

Female MRL/Mp mice were acquired from SLAC Laboratory Animal Co. (Shanghai, China) and housed at Changhai Hospital's Laboratory Animal Center, where they were kept under specific pathogen‐free conditions with a 12‐h light‐dark cycle. Each figure caption indicated the respective sample sizes. To control for environmental variations within the animal housing facility, the positions of the cages were rotated regularly. This ensured that factors such as light exposure, temperature variations, and other environmental influences were evenly distributed across all experimental groups. Mice were euthanized in compliance with the institutional ethical standards. All experimental procedures carried out in this study received formal approval from the Ethics Committee of the Laboratory Animal Center at Changhai Hospital. The approval, assigned the number CHEC‐2022‐M‐239, underscores the commitment to ethical standards and regulations regarding the treatment of laboratory animals throughout the research process. This ethical oversight ensures that the welfare of the animals involved is prioritized and that the experiments are conducted in accordance with established guidelines. The sample size was based on previous similar studies.^[^ ^77^ ^]^

### Induction of AIP

As per the manufacturer's guidelines, high molecular weight polyinosinic:polycytidylic acid (poly I:C, Cat# tlrl‐pic‐5, InvivoGen, CA, USA) was prepared to achieve a 1 mg mL⁻^1^ concentration. Female MRL/Mp mice, aged 6 weeks, received intraperitoneal injections of poly I:C at a dosage of 5 mg k^−1^g every three days over an 8‐week period, in line with previously documented procedures.^[^ ^78^ ^]^ The control group of mice was administered a 0.9% saline solution intraperitoneally. Then, all mice were sacrificed. Exogenous nampt was purchased from MedChemExpress LLC, USA (No. HY‐P70353) and FK866 was purchased from MedChemExpress LLC, USA (No. HY‐50876). For exploration of the efficacy of visfatin in vivo, nine female MRL/Mp mice were equally and randomly divided into three groups and AIP was induced as mentioned above. One group is the unexposed mice group (n  =  3), which received weekly intraperitoneal(IP) injections of normal saline, from week 4 to week 8, served as controls. For the other two groups, beginning in week 4, the mice received weekly intraperitoneal(IP) injections of either exogenous nampt, or FK866 (0.4 mg k^−1^g, n  =  3), from week 4 to week 8, and then, all mice were sacrificed and the pancreas was collected. The group allocation was performed by Dr. Haojie Huang and Dr. Deyu Zhang, who were the study coordinators and persons involved in the subsequent stages of the experiment.

### H&E and Masson Staining

The tissue slices assays were conducted utilizing the hematoxylin‐eosin (H&E) stain kit (Cat#G1120, Solarbio, China) and the Masson three‐color staining solution kit (Cat #G1006, Servicebio, China) according to the manufacturer's instructions.

### Flow Cytometry

PBMCs were isolated using density gradient centrifugation from blood drawn into EDTA tubes. The blood was diluted with Phosphate Buffered Saline (PBS) at a 1:1 ratio and rested for 30 min at room temperature, facilitating RBC sedimentation. The upper 8 mL lymphocyte and platelet‐rich plasma were then carefully transferred into a new tube, underlain with 6 mL Ficoll‐Paque Plus (density 1.077 g mL⁻^1^, GE Healthcare, USA), and centrifuged at 400 xg for 30 min at 23 °C without brake. The resultant buffy coat layer was aspirated and transferred to a clean tube. The PBMCs were washed twice using calcium and magnesium‐free PBS.

The cells were divided across five tubes to meet different staining requirements: Tube1 served as the blank control; Tubes 2, 3, and 4 were stained with PE‐CD45 (304007, Biolegend, USA), APC‐CD19 (302211, Biolegend, USA), and FOXP1 FITC (4402T, Cell Signaling Technology, USA) respectively; and Tube5 was stained with a combination of CD45, CD19, and FOXP1. Tubes 1 and 4 were resuspended in 100 µL PBS and temporarily stored in an ice box at 4 °C in the dark. Tubes 2, 3, and 5 were resuspended in 100 µL Staining Buffer, to which 2.5 µL of the respective antibodies were added and incubated in the dark for 60 min. Post‐incubation, all tubes were centrifuged at 500 g for 5 min, the supernatants were discarded, and cells were washed twice with Perm/Wash buffer. Subsequently, 1 mL of Fixation/Permeabilization solution (554714, BD Cytofix/Cytoperm, USA) was added to each tube and incubated at 4 °C for 20 min. After centrifugation and supernatant removal, the cells were washed twice more with Perm/Wash buffer. Tubes 1, 2, and 3 were then resuspended in 100 µL PBS and temporarily stored at 4 °C. Meanwhile, Tubes 4 and 5 were resuspended in 100 µL Staining Buffer, each receiving 2 µL of FOXP1 antibody and incubated in the dark for an additional 60 min. Post this second incubation, the cells were centrifuged, supernatants removed, and washed once with Perm/Wash buffer. Finally, 500 µL of PBS and 1 µL of fluorescent secondary antibody (FITC‐labeled goat anti‐rabbit IgG(H+L), A0562, Beyotime, China) were added, mixed, and incubated in the dark for 60 min. After final washes with Perm/Wash buffer, cells were resuspended in 100 µL PBS and analyzed using flow cytometry (FACSCalibur, BD Biosciences, USA).

### Schematic Drawing and Statistics

All experimental data processing was performed using R software 4.0.2 (The R Foundation for Statistical Computing, Vienna, Austria) and GraphPad Prism 8.01 software (GraphPad Company, San Diego, CA, USA). The results are shown as the mean± SD. The significance of differences between the two groups was determined with the t test and one‐way analysis of variance. Significance was accepted at a value of *p* < 0.05. The schematic diagram presented in Figure 8 was created using Biorender (available at https://app.biorender.com/), with the appropriate permissions obtained for its use. This online tool is specifically designed for generating high‐quality scientific illustrations, and we utilized it to accurately represent the concepts discussed in this work. The permission granted allows us to incorporate this diagram into this publication, ensuring that it adheres to the necessary copyright and ethical standards.

### Ethics Approval Statement

For human and mice ethics approval statement, ethical approval for this research was granted by the Ethics Committee or Laboratory Animal Center's Ethics Committee of Changhai Hospital, respectively (approval number: CHEC‐2022‐239 for human and CHEC‐2022‐M‐239 for mice).

## Conflict of Interest

The authors declare no conflict of interest.

## Supporting information



Supporting Information

## Data Availability

The data that support the findings of this study are openly available in GSA database at https://ngdc.cncb.ac.cn/, reference number 22200.

## References

[1] M. Masood , JGH Open 2022, 6, 3. 35071782 10.1002/jgh3.12688 PMC8762623

[2] E. C. Nista , S. S. De Lucia , V. Manilla , T. Schepis , A. Pellegrino , V. Ojetti , G. Pignataro , L. Zileri dal Verme , F. Franceschi , A. Gasbarrini , M. Candelli , Int. J. Mol. Sci. 2022, 23, 12667. 36293522 10.3390/ijms232012667 PMC9604056

[3] S. J. S. Nagpal , A. Sharma , C. S. T. A. Pancreatitis , Am. J. Gastroenterol. 2018, 113, 1301. 29910463 10.1038/s41395-018-0146-0

[4] K. Uchida , K. Okazaki , J. Gastroenterol. 2022, 57, 695. 35916965 10.1007/s00535-022-01891-7 PMC9522839

[5] H. Umehara , K. Okazaki , Y. Masaki , M. Kawano , M. Yamamoto , T. Saeki , S. Matsui , T. Yoshino , S. Nakamura , S. Kawa , H. Hamano , T. Kamisawa , T. Shimosegawa , A. Shimatsu , S. Nakamura , T. Ito , K. Notohara , T. Sumida , Y. Tanaka , T. Mimori , T. Chiba , M. Mishima , T. Hibi , H. Tsubouchi , K. Inui , H. Ohara , Mod. Rheumatol. 2011, 22, 21. 10.1007/s10165-011-0571-z 22218969

[6] H. Matsubayashi , H. Ishiwatari , K. Imai , Y. Kishida , S. Ito , K. Hotta , Y. Yabuuchi , M. Yoshida , N. Kakushima , K. Takizawa , N. Kawata , H. Ono , Int. J. Mol. Sci. 2019, 21, 257. 31905944 10.3390/ijms21010257 PMC6981453

[7] G. Katz , J. H. Stone , Annu. Rev. Med. 2022, 73, 545. 34669430 10.1146/annurev-med-050219-034449

[8] B. Chang Wu , J. Wlodarczyk , S. Nourmohammadi Abadchi , N. Shababi , J. L. Cameron , J. W. Harmon , eGastroenterology 2023, 1, e100014. 38292831 10.1136/egastro-2023-100014 PMC10827342

[9] A. F. Schier , Nat. Methods 2020, 17, 17. 31907464 10.1038/s41592-019-0693-3

[10] X. Tang , Y. Huang , J. Lei , H. Luo , X. Zhu , Cell Biosci. 2019, 9, 53. 31391919 10.1186/s13578-019-0314-y PMC6595701

[11] Y. Y. Tang , D. C. Wang , Y. Q. Wang , A. F. Huang , W. D. Xu , Front. Immunol. 2022, 13, 1073971. 36761171 10.3389/fimmu.2022.1073971 PMC9905447

[12] M. W. Luczak , C. Krawic , A. Zhitkovich , Cell Cycle 2021, 20, 1812. 34382917 10.1080/15384101.2021.1959988 PMC8525932

[13] D. Liu , L. Wang , W. Ha , K. Li , R. Shen , D. Wang , Chem.‐Biol. Interact. 2024, 387, 110808. 37980973 10.1016/j.cbi.2023.110808

[14] E. S. Carpenter , A. M. Elhossiny , P. Kadiyala , J. Li , J. McGue , B. D. Griffith , Y. Zhang , J. Edwards , S. Nelson , F. Lima , K. L. Donahue , W. Du , A. C. Bischoff , D. Alomari , H. R. Watkoske , M. Mattea , S. The , C. E. Espinoza , M. Barrett , C. J. Sonnenday , N. Olden , C.‐T. Chen , N. Peterson , V. Gunchick , V. Sahai , A. Rao , F. Bednar , J. Shi , T. L. Frankel , M. Pasca di Magliano , Cancer Discov. 2023, 13, 1324. 37021392 10.1158/2159-8290.CD-23-0013 PMC10236159

[15] M. Shiokawa , Y. Kodama , K. Kuriyama , K. Yoshimura , T. Tomono , T. Morita , N. Kakiuchi , T. Matsumori , A. Mima , Y. Nishikawa , T. Ueda , M. Tsuda , Y. Yamauchi , R. Minami , Y. Sakuma , Y. Ota , T. Maruno , A. Kurita , Y. Sawai , Y. Tsuji , N. Uza , K. Matsumura , T. Watanabe , K. Notohara , T. Tsuruyama , H. Seno , T. Chiba , Gut 2016, 65, 1322. 26964842 10.1136/gutjnl-2015-310336

[16] M. Shiokawa , Y. Kodama , K. Sekiguchi , T. Kuwada , T. Tomono , K. Kuriyama , H. Yamazaki , T. Morita , S. Marui , Y. Sogabe , N. Kakiuchi , T. Matsumori , A. Mima , Y. Nishikawa , T. Ueda , M. Tsuda , Y. Yamauchi , Y. Sakuma , T. Maruno , N. Uza , T. Tsuruyama , T. Mimori , H. Seno , T. Chiba , Sci. Transl. Med. 2018, 10, 997. 10.1126/scitranslmed.aaq0997 30089633

[17] H. Mattoo , J. H. Stone , S. Pillai , Autoimmunity 2017, 50, 19. 28166682 10.1080/08916934.2017.1280029 PMC5880292

[18] Y. Inoue , S. Nakayamada , S. Kubo , K. Yamagata , K. Sonomoto , S. Iwata , Y. Miyazaki , Y. Tanaka , Rheumatology 2022, 61, 3854. 34940835 10.1093/rheumatology/keab935

[19] B. C. Narmada , A. Khakpoor , N. Shirgaonkar , S. Narayanan , P. P. K. Aw , M. Singh , K. H. Ong , C. O. Owino , J. W. T. Ng , H. C. Yew , N. u. S. Binte Mohamed Nasir , V. B. Au , R. Sng , N. Kaliaperumal , H. H. T. W. Khine , F. C. di Tocco , O. Masayuki , S. Naikar , H. X. Ng , S. u. L. i. Chia , C. X. Y. i. Seah , M. H. j. Alnawaz , C. L. Y. Wai , A. Y. L. Tay , K. S. Mangat , V. Chew , W. Yu , J. E. Connolly , G. Periyasamy , et al., J. Hepatol. 2024, 81, 42. 38423478 10.1016/j.jhep.2024.02.017

[20] T. Watanabe , K. Minaga , K. Kamata , M. Kudo , W. Strober , Trends Immunol. 2018, 39, 874. 30401468 10.1016/j.it.2018.09.005

[21] N. Ishiguro , M. Moriyama , K. Furusho , S. Furukawa , T. Shibata , Y. Murakami , et al., Arthritis Rheumatol. 2020, 72, 166. 31339007 10.1002/art.41052 PMC6972995

[22] S. Furukawa , M. Moriyama , K. Miyake , H. Nakashima , A. Tanaka , T. Maehara , M. Iizuka‐Koga , H. Tsuboi , J.‐N. Hayashida , N. Ishiguro , M. Yamauchi , T. Sumida , S. Nakamura , Sci. Rep. 2017, 7, 42413. 28205524 10.1038/srep42413 PMC5304322

[23] L. Cheng , H. Yu , J. A. Wrobel , G. Li , P. Liu , Z. Hu , X. N. Xu , L. Su , JCI insight 2020, 5, e135344. 32406872 10.1172/jci.insight.135344 PMC7308046

[24] G. M. Seleznik , T. Reding , L. Peter , A. Gupta , S. G. Steiner , S. Sonda , C. S. Verbeke , E. Dejardin , I. Khatkov , S. Segerer , M. Heikenwalder , R. Graf , Gut 2018, 67, 1663. 28774888 10.1136/gutjnl-2016-313458

[25] C. V. Rift , L. C. Melchior , B. Kovacevic , P. Klausen , A. Toxværd , H. Grossjohann , J. G. Karstensen , L. Brink , H. Hassan , E. Kalaitzakis , J. Storkholm , D. Scheie , C. P. Hansen , E. L. Lund , P. Vilmann , J. P. Hasselby , Gastrointest. Endosc. 2023, 97, 50. 35964683 10.1016/j.gie.2022.08.008

[26] S. A. Rodriguez , S. D. Impey , C. Pelz , B. Enestvedt , G. Bakis , M. Owens , T. K. Morgan , Gastrointest. Endosc. 2016, 84, 252. 26808815 10.1016/j.gie.2016.01.042

[27] Y. Li , H. Song , X. Meng , R. Li , P. S. C. Leung , M. E. Gershwin , S. Zhang , S. Sun , J. Song , J. Autoimmun. 2023, 140, 103121. 37826920 10.1016/j.jaut.2023.103121

[28] T. Patzelt , S. J. Keppler , O. Gorka , S. Thoene , T. Wartewig , M. Reth , I. Förster , R. Lang , M. Buchner , J. Ruland , Proc. Natl. Acad. Sci. U. S. A. 2018, 115, 3120. 29507226 10.1073/pnas.1711335115 PMC5866538

[29] A. Masamune , K. Kikuta , S. Hamada , I. Tsuji , Y. Takeyama , T. Shimosegawa , K. Okazaki , J. Gastroenterol. 2020, 55, 462.31872350

[30] A. G. Lytle , J. E. Norton , C. L. Dorfmeier , S. Shen , J. P. McGettigan , J. Virol. 2013, 87, 9097. 23760241 10.1128/JVI.00800-13 PMC3754075

[31] R. Marzio , J. Mauël , S. Betz‐Corradin , Immunopharmacol. Immunotoxicol. 1999, 21, 565. 10466080 10.3109/08923979909007126

[32] Y. Li , Z. Wang , F. Han , M. Zhang , T. Yang , M. Chen , J. Du , Y. Wang , L. i. Zhu , H. Hou , Y. Chang , L. Han , X. Lyu , N. a. Zhang , W. Sun , Z. Cai , W. Wei , Ann. Rheum. Dis. 2023, 82, 1348. 37474274 10.1136/ard-2023-224363

[33] F. Wu , J. Gao , J. Kang , X. Wang , Q. Niu , J. Liu , L. Zhang , Front. Immunol. 2021, 12, 750753. 34650569 10.3389/fimmu.2021.750753 PMC8505880

[34] P. Mota , V. Reddy , D. Isenberg , Expert Rev. Clin. Immunol. 2017, 13, 667. 27841031 10.1080/1744666X.2017.1259068

[35] T. Cargill , M. Makuch , R. Sadler , L. C. Lighaam , R. Peters , M. van Ham , P. Klenerman , A. Bateman , T. Rispens , E. Barnes , E. L. Culver , Clin. Transl. Gastroenterol. 2019, 10, e00020. 31033594 10.14309/ctg.0000000000000020 PMC6602789

[36] K. Okazaki , K. Sumimoto , T. Mitsuyama , K. Uchida , Journal of the Japanese Society of Clinical Immunology Japan 2014, 37, 11. 10.2177/jsci.37.11 24598063

[37] C. G. Vinuesa , M. A. Linterman , D. Yu , I. C. MacLennan , Annu. Rev. Immunol. 2016, 34, 335. 26907215 10.1146/annurev-immunol-041015-055605

[38] D. A. Rao , M. F. Gurish , J. L. Marshall , K. Slowikowski , C. Y. Fonseka , Y. Liu , L. T. Donlin , L. A. Henderson , K. Wei , F. Mizoguchi , N. C. Teslovich , M. E. Weinblatt , E. M. Massarotti , J. S. Coblyn , S. M. Helfgott , Y. C. Lee , D. J. Todd , V. P. Bykerk , S. M. Goodman , A. B. Pernis , L. B. Ivashkiv , E. W. Karlson , P. A. Nigrovic , A. Filer , C. D. Buckley , J. A. Lederer , S. Raychaudhuri , M. B. Brenner , Nature 2017, 542, 110. 28150777 10.1038/nature20810 PMC5349321

[39] K. Tenbrock , T. Rauen , Clin. Immunol. 2022, 239, 109031. 35526790 10.1016/j.clim.2022.109031

[40] X. Wei , X. Niu , J. Autoimmun. 2023, 134, 102976. 36525939 10.1016/j.jaut.2022.102976

[41] X. Zhang , R. Ge , H. Chen , M. Ahiafor , B. Liu , J. Chen , X. Fan , Mediators Inflamm. 2021, 2021, 2058964. 34552387 10.1155/2021/2058964 PMC8452443

[42] X. M. Meng , D. J. Nikolic‐Paterson , H. Y. Lan , Nat. Rev. Nephrol. 2016, 12, 325. 27108839 10.1038/nrneph.2016.48

[43] J. Massagué , D. Sheppard , Cell 2023, 186, 4007. 37714133 10.1016/j.cell.2023.07.036 PMC10772989

[44] E. Batlle , J. Massagué , Immunity 2019, 50, 924. 30995507 10.1016/j.immuni.2019.03.024 PMC7507121

[45] T. Zhuang , M.‐H. Chen , R.‐X. i. Wu , J. Wang , X. i.‐D. e. Hu , T. Meng , A. i.‐H. Wu , Y. Li , Y.‐F. Yang , Y. u. Lei , D.‐H. Hu , Y.‐X. Li , L. i. Zhang , A. i.‐J. Sun , W. Lu , G.‐N. Zhang , J.‐L. i. Zuo , C.‐C. Ruan , Nat. Commun. 2024, 15, 1995. 38443404 10.1038/s41467-024-46357-x PMC10914760

[46] B. Keith , R. S. Johnson , M. C. Simon , Nat. Rev. Cancer 2011, 12, 9. 22169972 10.1038/nrc3183 PMC3401912

[47] K. Kamata , T. Watanabe , K. Minaga , A. Hara , T. Yoshikawa , A. Okamoto , K. Yamao , M. Takenaka , A. h.‐M. Park , M. Kudo , Int. Immunol. 2019, 31, 795. 31287532 10.1093/intimm/dxz050

[48] T. Yoshikawa , K. Minaga , A. Hara , I. Sekai , M. Kurimoto , Y. Masuta , Y. Otsuka , R. Takada , K. Kamata , A. h.‐M. Park , S. Takamura , M. Kudo , T. Watanabe , Int. Immunol. 2022, 34, 621. 36044992 10.1093/intimm/dxac039

[49] S. E. Corcoran , L. A. O'Neill , J. Clin. Invest. 2016, 126, 3699. 27571407 10.1172/JCI84431 PMC5096812

[50] D. Wang , S. Han , G. Lv , Y. Hu , W. Zhuo , Z. Zeng , J. Tang , Y. Huang , F. Wang , J. Wang , Y. Zhao , G. Zhao , Gastroenterology 2023, 165, 1488. 37634735 10.1053/j.gastro.2023.08.029

[51] B. Samal , Y. Sun , G. Stearns , C. Xie , S. Suggs , I. McNiece , Mol. Cell. Biol. 1994, 14, 1431. 8289818 10.1128/mcb.14.2.1431-1437.1994 PMC358498

[52] J. R. Revollo , A. A. Grimm , S. Imai , Curr. Opin. Gastroenterol. 2007, 23, 164. 17268245 10.1097/MOG.0b013e32801b3c8f

[53] F. Carbone , L. Liberale , A. Bonaventura , A. Vecchiè , M. Casula , M. Cea , Compr. Physiol. 2017, 7, 603. 28333382 10.1002/cphy.c160029

[54] C. A. Curat , V. Wegner , C. Sengenès , A. Miranville , C. Tonus , R. Busse , A. Bouloumié , Diabetologia 2006, 49, 744. 16496121 10.1007/s00125-006-0173-z

[55] S. Ognjanovic , T. L. Ku , G. D. Bryant‐Greenwood , Am. J. Obstet. Gynecol. 2005, 193, 273. 16021090 10.1016/j.ajog.2004.11.003 PMC1382169

[56] M. Gosset , F. Berenbaum , C. Salvat , A. Sautet , A. Pigenet , K. Tahiri , C. Jacques , Arthritis Rheum. 2008, 58, 1399. 18438860 10.1002/art.23431

[57] Y. u. J. Heo , S.‐E. Choi , N. Lee , J. a. Y. Jeon , S. J. Han , D. J. Kim , Y. Kang , K. W. Lee , H. J. Kim , J. Gastroenterol. Hepatol. 2021, 36, 2592. 33600604 10.1111/jgh.15465

[58] C. Shen , R. Fang , J. Wang , N. Wu , S. Wang , T. Shu , J. Dai , M. Feng , X. Chen , J. Cell. Mol. Med. 2023, 27, 2562. 37584247 10.1111/jcmm.17854 PMC10468652

[59] E. Franco‐Trepat , A. Alonso‐Pérez , M. Guillán‐Fresco , A. Jorge‐Mora , O. Gualillo , J. J. Gómez‐Reino , R. Gómez Bahamonde , Expert Opin. Ther. Targets 2019, 23, 607. 31074669 10.1080/14728222.2019.1617274

[60] H. S. Farghaly , K. A. Metwalley , F.‐A. Ahmed , D. M. Raafat , O. El‐Asheer , A. M. Ali , A. Bahdawy , A. M. Zahran , Ther. Adv. Endocrinol. Metab. 2017, 8, 119. 28979761 10.1177/2042018817731073 PMC5617091

[61] K. J. Aney , W.‐J. Jeong , A. F. Vallejo , C. Burdziak , E. Chen , A. Wang , P. Koak , K. Wise , K. Jensen , D. Pe'er , S. K. Dougan , L. Martelotto , S. Nissim , Gastroenterology 2024, 166, 1100. 38325760 10.1053/j.gastro.2024.01.043 PMC11102849

[62] K. Okazaki , S. Kawa , T. Kamisawa , T. Ikeura , T. Itoi , T. Ito , K. Inui , A. Irisawa , K. Uchida , H. Ohara , K. Kubota , Y. Kodama , K. Shimizu , R. Tonozuka , T. Nakazawa , T. Nishino , K. Notohara , Y. Fujinaga , A. Masamune , H. Yamamoto , T. Watanabe , T. Nishiyama , M. Kawano , K. Shiratori , T. Shimosegawa , Y. Takeyama , J. Gastroenterol. 2022, 57, 225. 35192048 10.1007/s00535-022-01857-9 PMC8938398

[63] H. J. Asbun , K. Conlon , L. Fernandez‐Cruz , H. Friess , S. V. Shrikhande , M. Adham , C. Bassi , M. Bockhorn , M. Büchler , R. M. Charnley , C. Dervenis , A. Fingerhutt , D. J. Gouma , W. Hartwig , C. Imrie , J. R. Izbicki , K. D. Lillemoe , M. Milicevic , M. Montorsi , J. P. Neoptolemos , A. A. Sandberg , M. Sarr , C. Vollmer , C. J. Yeo , L. W. Traverso , Surgery 2014, 155, 887. 24661765 10.1016/j.surg.2013.12.032

[64] S. Nikolic , P. Ghorbani , R. Pozzi Mucelli , S. Ghazi , F. Baldaque‐Silva , M. Del Chiaro , E. Sparrelid , C. S. Verbeke , J.‐M. Löhr , M. Vujasinovic , Dig. Surg. 2022, 39, 32. 34915509 10.1159/000521490 PMC8985041

[65] T. Shimosegawa , S. T. Chari , L. Frulloni , T. Kamisawa , S. Kawa , M. Mino‐Kenudson , M.‐H. Kim , G. Klöppel , M. M. Lerch , M. Löhr , K. Notohara , K. Okazaki , A. Schneider , L. Zhang , Pancreas 2011, 40, 352. 21412117 10.1097/MPA.0b013e3182142fd2

[66] Y. Hao , S. Hao , E. Andersen‐Nissen , W. M. Mauck , S. Zheng , A. Butler , M. J. Lee , A. J. Wilk , C. Darby , M. Zager , P. Hoffman , M. Stoeckius , E. Papalexi , E. P. Mimitou , J. Jain , A. Srivastava , T. Stuart , L. M. Fleming , B. Yeung , A. J. Rogers , J. M. McElrath , C. A. Blish , R. Gottardo , P. Smibert , R. Satija , Cell 2021, 184, 3573. 34062119 10.1016/j.cell.2021.04.048 PMC8238499

[67] M. D. Young , S. Behjati , GigaScience 2020, 9, giaa151. 33367645 10.1093/gigascience/giaa151 PMC7763177

[68] C. S. McGinnis , L. M. Murrow , G. Z. J. DoubletFinder , Cell syst. 2019, 8, 329. 30954475 10.1016/j.cels.2019.03.003 PMC6853612

[69] I. Korsunsky , N. Millard , J. Fan , K. Slowikowski , F. Zhang , K. Wei , Y. Baglaenko , M. Brenner , P. o.‐R. u. Loh , S. Raychaudhuri , Nat. Methods 2019, 16, 1289. 31740819 10.1038/s41592-019-0619-0 PMC6884693

[70] A. A. Sergushichev , An algorithm for fast preranked gene set enrichment analysis using cumulative statistic calculation 2016, 060012.

[71] J. Cao , M. Spielmann , X. Qiu , X. Huang , D. M. Ibrahim , A. J. Hill , F. Zhang , S. Mundlos , L. Christiansen , F. J. Steemers , C. Trapnell , J. Shendure , Nature 2019, 566, 496. 30787437 10.1038/s41586-019-0969-x PMC6434952

[72] S. Jin , C. F. Guerrero‐Juarez , L. Zhang , I. Chang , R. Ramos , C.‐H. Kuan , P. Myung , M. V. Plikus , Q. Nie , Nat. Commun. 2021, 12, 1088. 33597522 10.1038/s41467-021-21246-9 PMC7889871

[73] E. Dann , N. C. Henderson , S. A. Teichmann , M. D. Morgan , J. C. Marioni , Nat. Biotechnol. 2022, 40, 245. 34594043 10.1038/s41587-021-01033-z PMC7617075

[74] S. Aibar , C. B. González‐Blas , T. Moerman , V. A. Huynh‐Thu , H. Imrichova , G. Hulselmans , F. Rambow , J.‐C. Marine , P. Geurts , J. Aerts , J. van den Oord , Z. K. Atak , J. Wouters , S. Aerts , Nat. Methods 2017, 14, 1083. 28991892 10.1038/nmeth.4463 PMC5937676

[75] R. F. Hwang , T. Moore , T. Arumugam , V. Ramachandran , K. D. Amos , A. Rivera , B. Ji , D. B. Evans , C. D. Logsdon , Cancer Res. 2008, 68, 918. 18245495 10.1158/0008-5472.CAN-07-5714 PMC2519173

[76] H. Yin , Z. Zhang , D. Zhang , L. Peng , C. Xia , X. Yang , X. Wang , Z. Li , J. Chang , H. Huang , J. Mater. Chem. B 2023, 11, 9163. 37642526 10.1039/d3tb01287e

[77] J. Yang , H. Wei , Y. Lin , E. S. H. Chu , Y. Zhou , H. Gou , S. Guo , H. C. H. Lau , A. H. K. Cheung , H. Chen , K. a. F. To , J. J. Y. Sung , Y. Wang , J. Yu , Gastroenterology 2024, 166, 323. 37858797 10.1053/j.gastro.2023.10.012

[78] T. Schwaiger , C. van den Brandt , B. Fitzner , S. Zaatreh , F. Kraatz , A. Dummer , H. Nizze , M. Evert , B. M. Bröker , M. C. Brunner‐Weinzierl , T. Wartmann , T. Salem , M. M. Lerch , R. Jaster , J. Mayerle , Gut 2014, 63, 494. 23564336 10.1136/gutjnl-2012-303635

