# Supplementary material for: Unraveling the Microenvironment and the Pathogenic Axis of HIF‐1α–Visfatin–Fibrosis in Autoimmune Pancreatitis Using a Single‐Cell Atlas
Source: Adv Sci (Weinh). 2025 Jan 31;12(12):2412282. doi: 10.1002/advs.202412282 (PMC11948021; doi:10.1002/advs.202412282)
Supplement: Supplementary file 1 — Supporting Information [file ADVS-12-2412282-s001.docx]

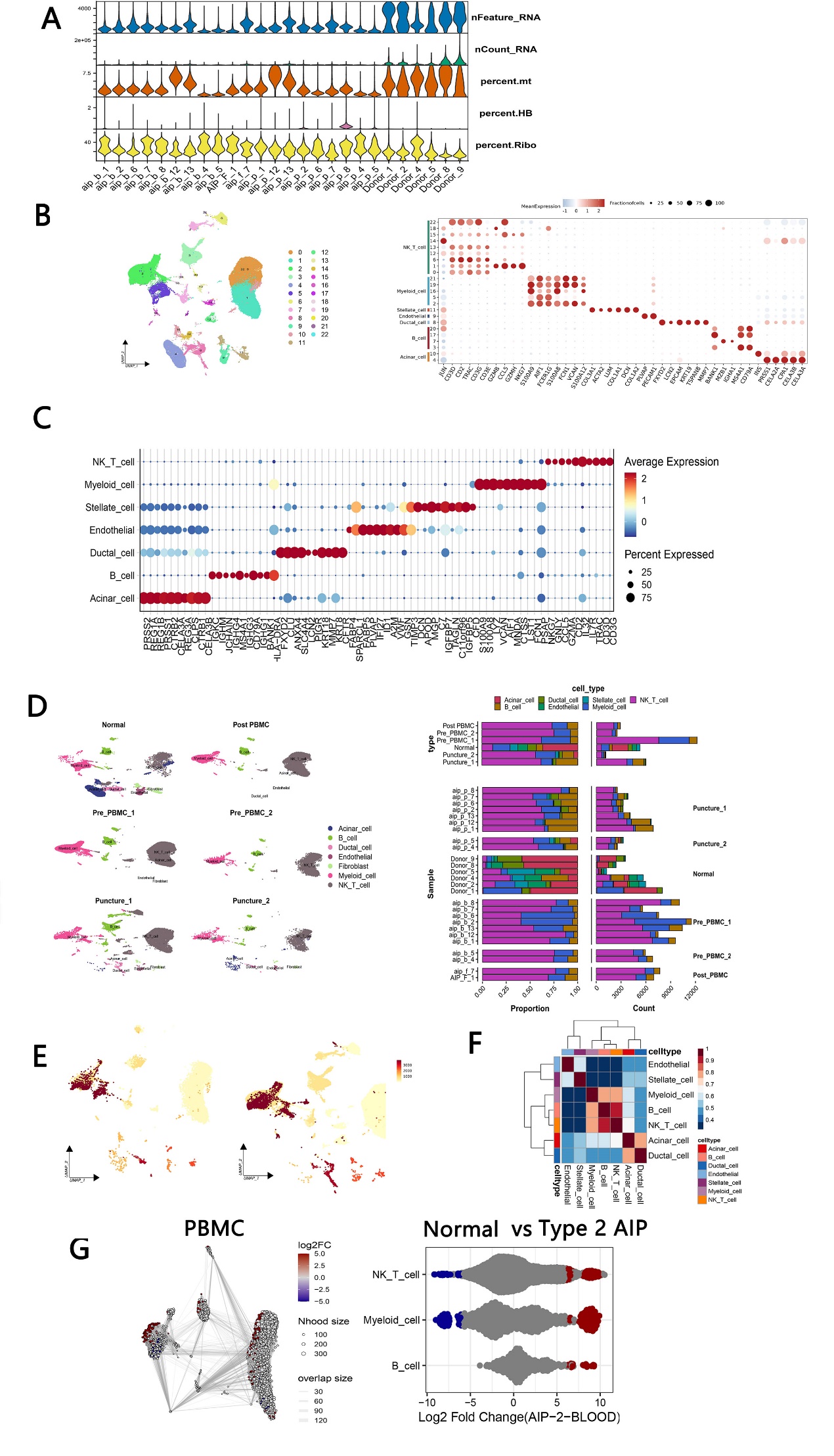


Figure S1 (A) The counts of total genes, features, and mitochondrial genes per cell in each sample. (B) Left: The UMAP plot of each cluster in PBMC data before annotation. Right: The dotplot of marker genes in each cluster. (C) The dotplot of top 10 expressed genes in each cellgroup. (D) Left: The UMAP plot of each cell cluster in PBMC data before annotation, divided by sample group. Right: The propotion or count of each celltype in each samples or group. (E) UMAP plot shows the number of DEGs between the AIP group and normal group(Left), or post-PBMC group and normal group (Right). (F) Heatmap of the correlation analysis of each cell group. (G) Differential abundance UMAP and beeswarm plots from PBMCs between Normal group and Type 2 AIP groups by cell type. Each dot is a neighbourhood of cells calculated using miloR. Neighbourhoods that reach significance (spatial FDR < 0.05) and are coloured by log fold-change.


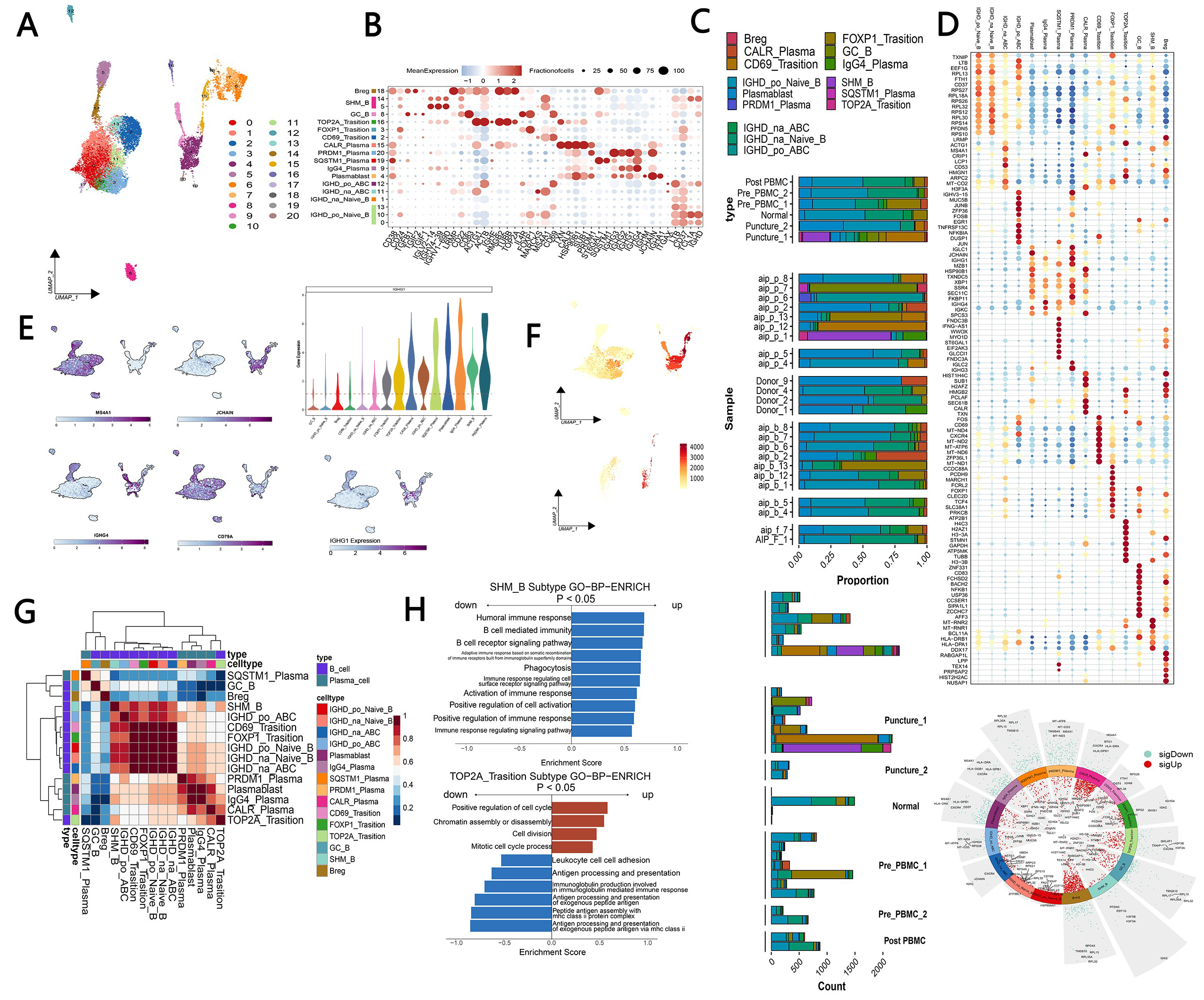


Figure S2 (A) The UMAP plot of each B cell cluster before annotation, divided by sample group. (B) The dotplot of marker genes in each cluster. (C) The propotion or count of each celltype in each samples or group. (D) Up: The dotplot of top 10 overexpressed genes in each cell subgroup. Down: The pie plot of top 5 overexpressed genes and 5 downexpressed genes in each cell subgroup. (E) UMAP plot shows the expression and distribution of *MS4A1*, *JCHAIN*, *IGHG4*, *IGHG1, CD79A*. (F) UMAP plot shows the number of DEGs between the AIP group and normal group(Left), or post-PBMC group and normal group (Right). (G) Heatmap of the correlation analysis of each cell subgroup. (H) The representative Gene Ontology terms for SHM B cells, TOP2A Transition B cells in the type 1 AIP group compared to those in the normal pancreas or post-PBMC group according to GSEA.


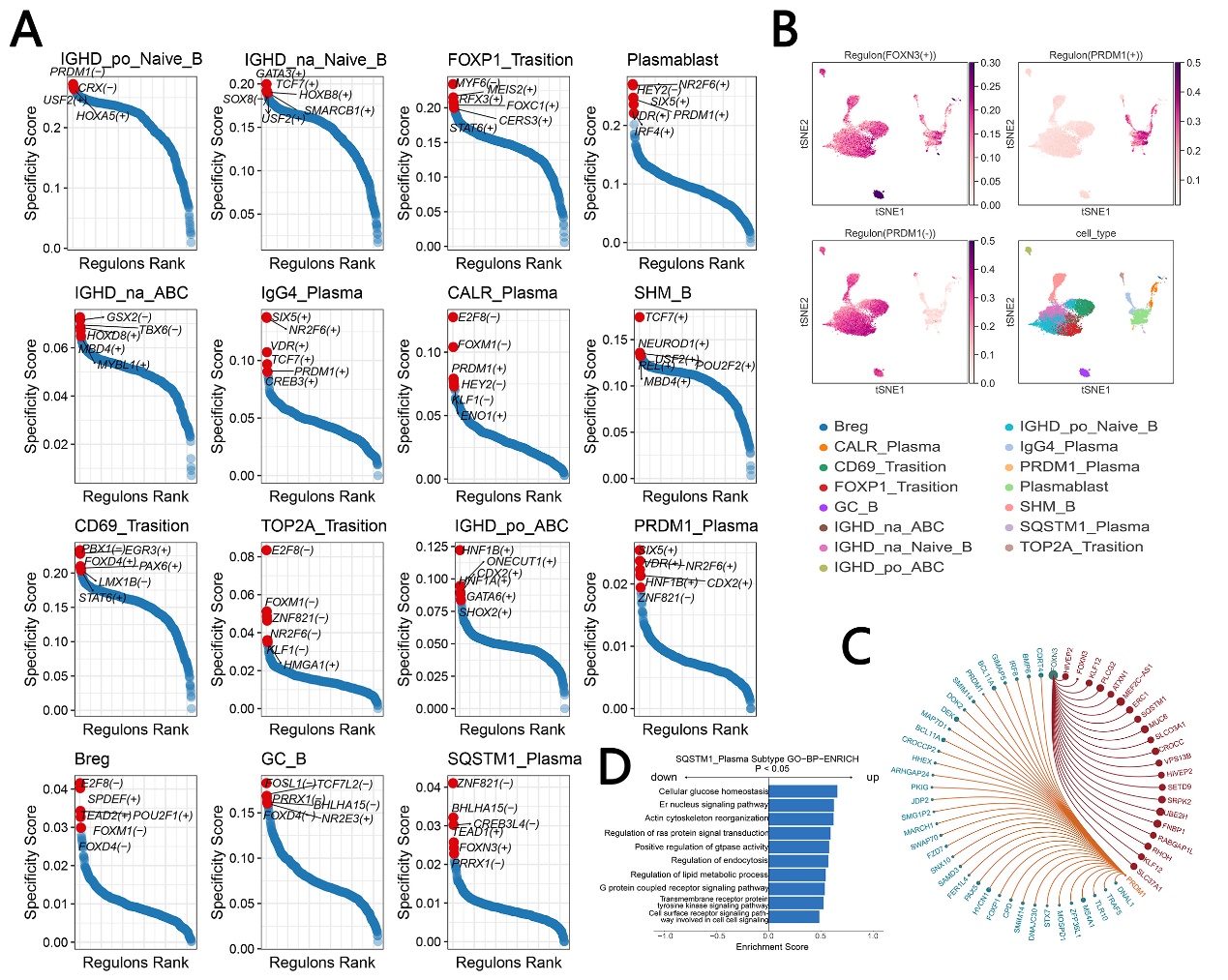


Figure S3 (A) The score of transcriptional factor in each B cell type. (B) The distribution of two putative transcriptional factor, including FOXN3, PRDM1. (C) The potential interaction between PRDM1 and FOXN3 and other high expressed transcriptional factors.


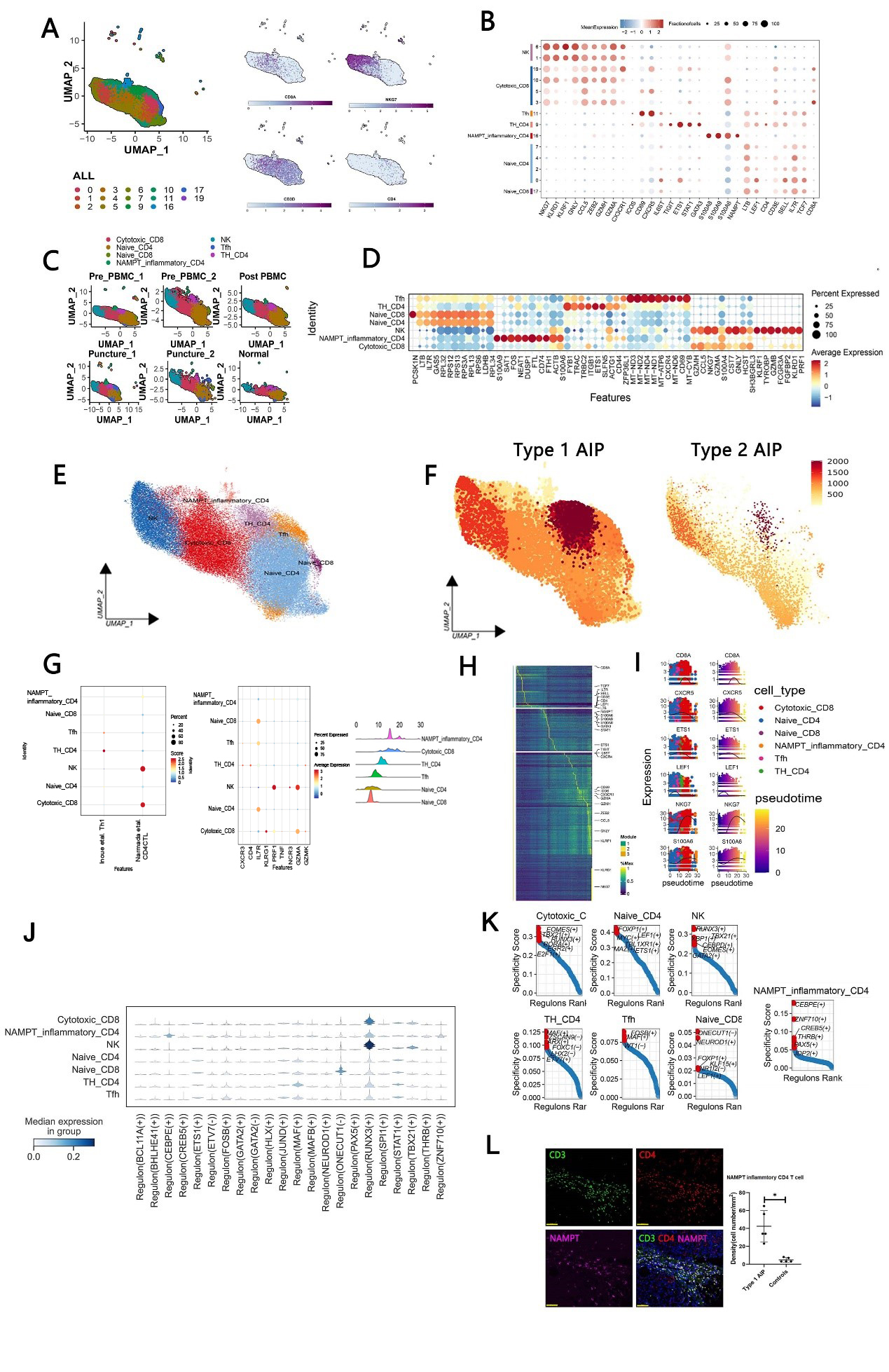


Figure S4 (A) Left: the UMAP plot of each T cell cluster before annotation. Right: UMAP plot shows the expression and distribution of *CD8A*, *NKG7*, *CD3D* and *CD4*. (B) The dotplot of marker genes in each cluster. (C) The UMAP plot of each T cell cluster after annotation, divided by sample group. (D) The dotplot of top 10 overexpressed genes in each cell subgroup. (E) The UMAP plot of each T cell cluster after annotation. (F) UMAP plot shows the number of DEGs between the type 1 AIP group and normal group(Left), or the type 2 AIP group and normal group(Left). (G) Left: The expression of Th1 gene set from Y. Inoue et al and CD4 CTL gene set from B.C. Narmada et al. Middle: The expression of markers from Th1 and CD4 CTL cells in each T cell subtype. Right: The pseudotime analysis of each T-cell subgroup in type 1 AIP pancreatic lesions. (H) DEGs and modules based on pseudotime analysis. (I) The expression of several cell markers over pseudotime. (J) The expression of significant transcriptional factor in each T cell subtype. (K) The score of transcriptional factor in each T cell type. (L) Right: Representative immunofluorescence image of NAMPT-positive inflammatory CD4+ T-cells in pancreatic lesions of type 1 AIP patients. Left: The density of NAMPT-positive inflammatory CD4+ T-cells in the pancreatic tissues of type 1 AIP patients (n=5) and controls (n=5) (*p < 0.05).


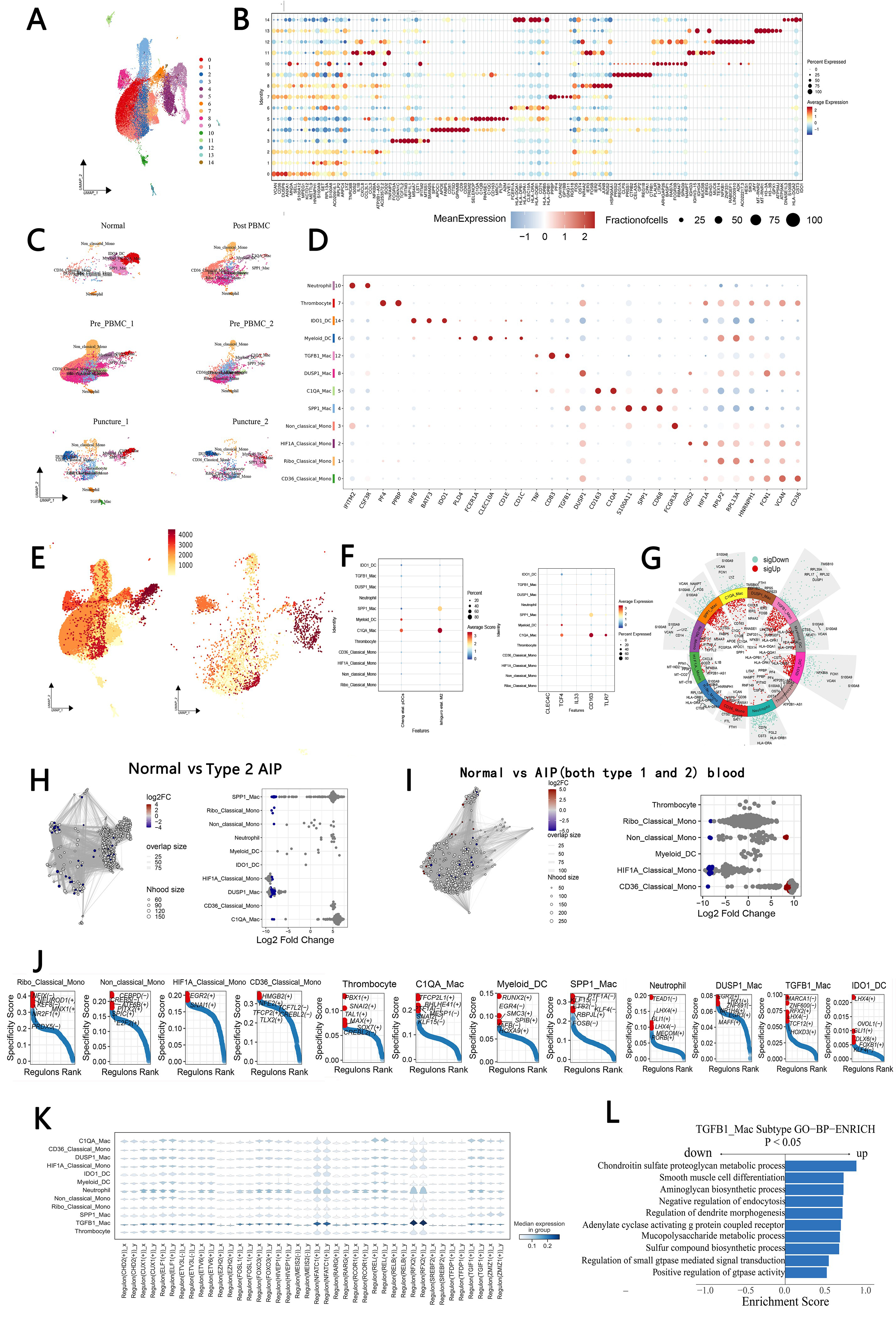


Figure S5 (A) UMAP plot of each myeloid cell cluster before annotation. Right: UMAP plot shows the expression and distribution of *CD8A*, *NKG7*, *CD3D* and *CD4*. (B) The dotplot of top 10 overexpressed genes in each cell subgroup. (C) The UMAP plot of each B cell cluster after annotation, divided by sample group. (D) The dotplot of marker genes in each cluster. (E) Left: UMAP plot shows the number of DEGs between the type 1 AIP group and normal group. Right: UMAP plot shows the number of DEGs between the type 2 AIP group and normal group. (F) Left: The expression of pDCs gene set from Cheng et al and M2 macrophage gene sets from Ishiguro. Right: The expression of markers from pDCs and M2 macrophage gene sets in each macrophage cell subtype. (G) The pie plot of top 5 overexpressed genes and 5 downexpressed genes in each cell subgroup. (H) Differential abundance UMAP and beeswarm plots from PBMCs between Normal group and Type 2 AIP groups by cell type. Each dot is a neighbourhood of cells calculated using miloR. Neighbourhoods that reach significance (spatial FDR < 0.05) and are coloured by log fold-change. (I) Differential abundance UMAP and beeswarm plots from PBMCs between Normal group and type 1 and type 2 AIP groups by cell type. Each dot is a neighbourhood of cells calculated using miloR. Neighbourhoods that reach significance (spatial FDR < 0.05) and are coloured by log fold-change. (J) The score of transcriptional factor in each myeloid cell subgroup. (K) The expression of significant transcriptional factor in each myeloid cell subgroup. (L) The representative Gene Ontology terms for *TGFB1* macrophages in the type 1 and type 2 AIP group compared to those in the normal pancrea group according to GSEA.

Figure S6 (A) The number of inferred interactions among normal group, type 2 AIP group and type 1 AIP group. (B) The incoming interaction strength among normal group, type 2 AIP group and type 1 AIP group. (C) The intereaction numbers between IgG4 plasma cell and the others in type 1 AIP, type 2 AIP and normal pancreas. (D) The intereaction numbers between Acinar cell and the others in type 1 AIP, type 2 AIP and normal pancreas. (E) The heatmap of intereaction numbers among all cell subgroups in type 1 AIP, type 2 AIP and normal pancreas. (F) The changed ligand pairs between others and fibroblast(pancreatic stellar cells) in type 1 AIP group. (G) The changed ligand pairs between others and fibroblast(pancreatic stellar cells) in type 2 AIP group. Red frame: Visfatin related ligand pairs.


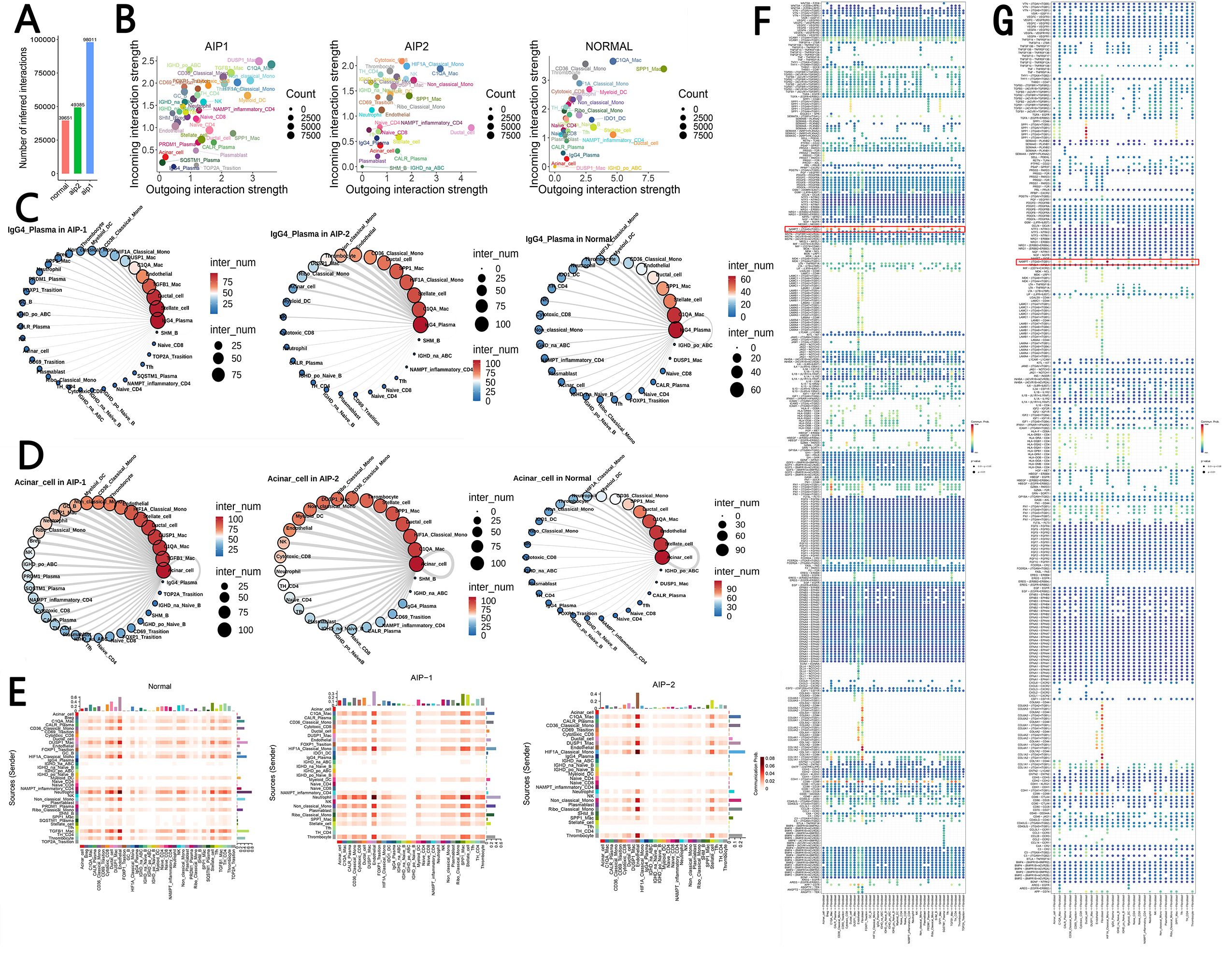


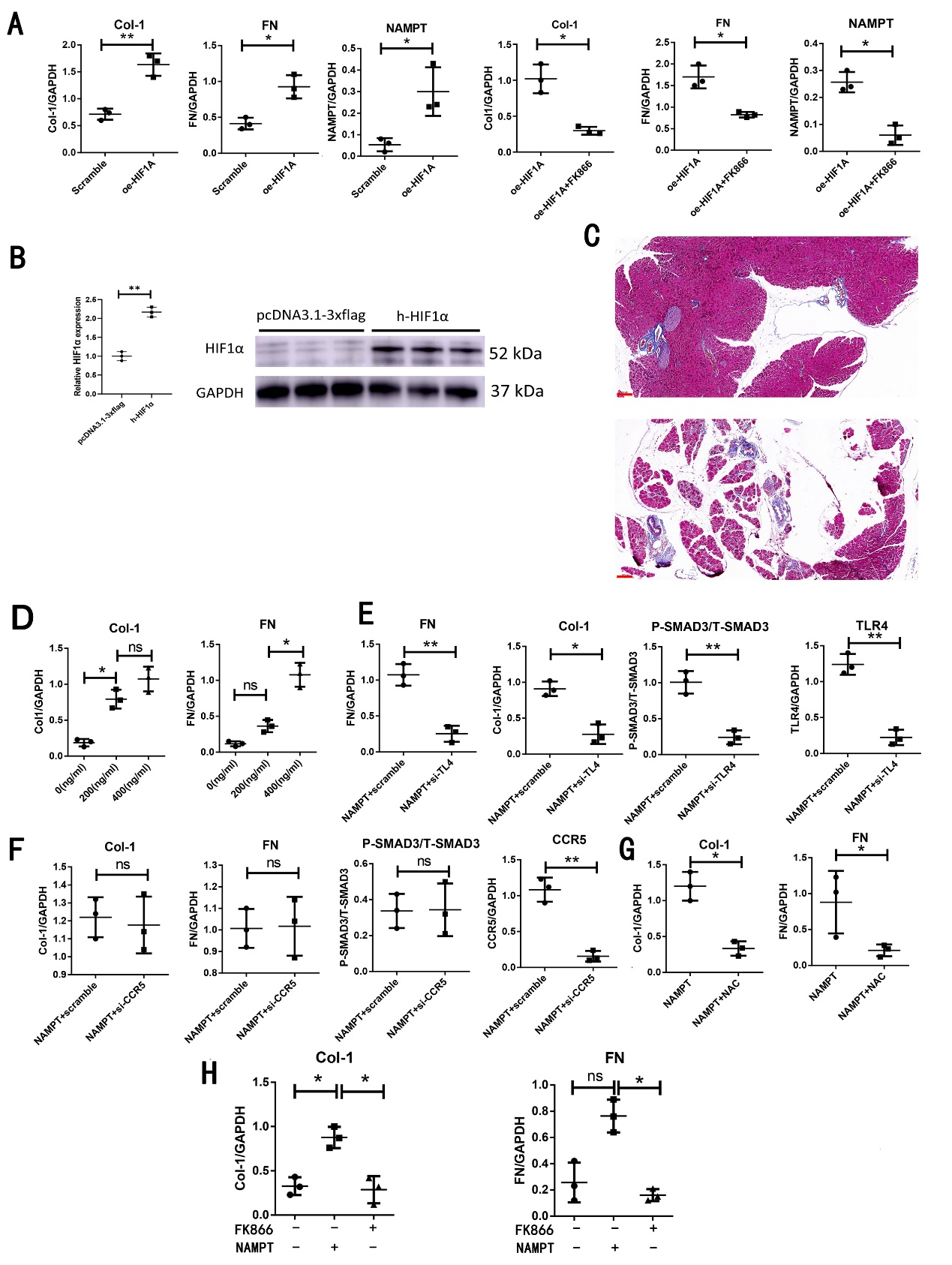


Figure S7 (A) The relative density of collagen-1 (col-1), fibronectin (Fn) and NAMPT between scamble control group and oe-HIF1A group, or between oe-HIF1A and oe-HIF1A+FK866 group in MPSC. (B) Left: the relative expression of *HIF1A* between pcDNA3.1-3xflag group and 293 T cells with high expression of *HIF1A*. Right: the expression of HIF-1α and GAPDH between pcDNA3.1-3xflag group and 293 T cells with high expression of *HIF1A* in protein level. (C) Upper: The representative imagine of pancreas from normal mice. Down: The representative imagine of pancreas from AIP mice model. (D) The relative density of protein expression levels of collagen-1 (Col-1), fibronectin (FN), and GAPDH after stimulation with exogenous NAMPT. (E) The relative density of protein expression levels of collagen-1 (Col-1), fibronectin (FN), phosphorylated SMAD3 (p-SMAD3), total SMAD3 (T-SMAD3) and GAPDH in the HPSC after group stimulated with exogenous NAMPT in the scramble control group and TLR4 knockdown (si-TLR4) group. (F) The relative density of protein expression levels of collagen-1 (Col-1), fibronectin (FN), phosphorylated SMAD3 (p-SMAD3), total SMAD3 (T-SMAD3) and GAPDH after stimulation with exogenous NAMPT in the scramble control group and the CCR5 knockdown (si-CCR5) group. (G) The relative density of protein expression levels of collagen-1 (Col-1), fibronectin (FN) and GAPDH in HPSCs after stimulation with exogenous NAMPT in the scramble control group and TLR4 knockdown (si-TLR4) group. (H) The relative density of protein expression levels of collagen-1 (Col-1), fibronectin (FN) and GAPDH in cells stimulated with or without exogenous NAMPT or FK866. * indicates p<0.05, ** indicates p<0.01, *** indicates p<0.001, **** indicates p<0.0001.

Table S1. The patients’ information of each samples enrolled in single-cell sequencing.

| Number | Source | Gender | Age(y/o) | IgG4(g/L) | Pnacreas samples(after 1 day) | Location | Diagnosis（I/II) | Blood samples(after 1 day) | Blood samples(after 3 month) |
| --- | --- | --- | --- | --- | --- | --- | --- | --- | --- |
| 1 | Tissue and peripheral blood single cell sequencing | Male | 60 | 20.5 | AIP-P-1 | Tail | Type I autoimmune pancreatitis | AIP-b-1 | AIP-f-1 |
| 2 | Tissue and peripheral blood single cell sequencing | Male | 63 | 15.1 | AIP-P-2 | Head | Type I autoimmune pancreatitis | AIP-b-2 |  |
| 3 | Tissue and peripheral blood single cell sequencing | Male | 53 | 1.08 | AIP-P-4 | Head | Type II autoimmune pancreatitis | AIP-b-4 |  |
| 4 | Tissue and peripheral blood single cell sequencing | Female | 32 | 0.01 | AIP-P-5 | Head | Type II autoimmune pancreatitis | AIP-b-5 |  |
| 5 | Tissue and peripheral blood single cell sequencing | Male | 63 | 7.64 | AIP-P-6 | Body | Type I autoimmune pancreatitis | AIP-b-6 |  |
| 6 | Tissue and peripheral blood single cell sequencing | Male | 33 | 7.98 | AIP-P-7 | Body | Type I autoimmune pancreatitis | AIP-b-7 | AIP-f-7 |
| 7 | Tissue and peripheral blood single cell sequencing | Male | 63 | 3 | AIP-P-8 | Tail | Type I autoimmune pancreatitis | AIP-b-8 |  |
| 8 | Tissue and peripheral blood single cell sequencing | Male | 65 | 2.28 | aip-p-12 | Head | Type I autoimmune pancreatitis | aip-b-12 |  |
| 9 | Tissue and peripheral blood single cell sequencing | Male | 77 | 6.03 | aip-p-13 | Head | Type I autoimmune pancreatitis | aip-b-13 |  |

Table S2. The patients’ information of each pancreatic lesion enrolled in immunofluorescence, and immunohistochemistry

| Number | Gender | Age(y) | IgG4(g/L) | CA199(u/ml) | Diagnosis |
| --- | --- | --- | --- | --- | --- |
| 1 | Male | 53 | - | 43.4 | intraductal papillary mucinous neoplasm(para-tumor tissue) |
| 2 | Male | 57 | - | 103.7 | intraductal papillary mucinous neoplasm(para-tumor tissue) |
| 3 | Female | 65 | - | 88.4 | intraductal papillary mucinous neoplasm(para-tumor tissue) |
| 4 | Female | 57 | - | 65.3 | intraductal papillary mucinous neoplasm(para-tumor tissue) |
| 5 | Male | 53 | - | 87.6 | intraductal papillary mucinous neoplasm(para-tumor tissue) |
| 6 | Male | 45 | 8.38 | 53.5 | Type I autoimmune pancreatitis (Postoperative pathological diagnosis) |
| 7 | Female | 37 | 9.12 | 182.4 | Type I autoimmune pancreatitis (Postoperative pathological diagnosis) |
| 8 | Male | 43 | 10.45 | 89.5 | Type I autoimmune pancreatitis  (Postoperative pathological diagnosis) |
| 9 | Male | 76 | 11.32 | 56.7 | Type I autoimmune pancreatitis  (Postoperative pathological diagnosis) |
| 10 | Female | 47 | 2.21 | 128.3 | Type I autoimmune pancreatitis (Postoperative pathological diagnosis) |
| 11 | Male | 56 | 1.16 | 173.2 | Type II autoimmune pancreatitis (Postoperative pathological diagnosis) |
| 12 | Female | 43 | 0.13 | 193.5 | Type II autoimmune pancreatitis (Postoperative pathological diagnosis) |
| 13 | Male | 37 | 0.62 | 93.5 | Type II autoimmune pancreatitis (Postoperative pathological diagnosis) |
| 14 | Male | 56 | 0.12 | 72.9 | Type II autoimmune pancreatitis (Postoperative pathological diagnosis) |
| 15 | Female | 34 | 0.05 | 75.2 | Type II autoimmune pancreatitis (Postoperative pathological diagnosis) |

Table S3. The information of each individuals enrolled in flow cytometry and elisa.

| Number | Gender | Age(y) | IgG4(g/L) | Diagnosis | Tissue character |
| --- | --- | --- | --- | --- | --- |
| 1 | Male | 54 | 8.6 | Type I autoimmune pancreatitis | Plasma and PBMC |
| 2 | Male | 65 | 8.5 | Type I autoimmune pancreatitis | Plasma and PBMC |
| 3 | Female | 45 | 6.3 | Type I autoimmune pancreatitis | Plasma and PBMC |
| 4 | Male | 58 | 11.3 | Type I autoimmune pancreatitis | Plasma and PBMC |
| 5 | Female | 47 | 10.3 | Type I autoimmune pancreatitis | Plasma and PBMC |
| 6 | Male | 58 | 0.5 | Type II autoimmune pancreatitis | Plasma and PBMC |
| 7 | Male | 46 | 1 | Type II autoimmune pancreatitis | Plasma and PBMC |
| 8 | Female | 74 | 1.2 | Type II autoimmune pancreatitis | Plasma and PBMC |
| 9 | Female | 71 | 0.9 | Type II autoimmune pancreatitis | Plasma and PBMC |
| 10 | Male | 61 | 0.73 | Type II autoimmune pancreatitis | Plasma and PBMC |
| 11 | Male | 42 | - | Healthy donor | Plasma and PBMC |
| 12 | Female | 54 | - | Healthy donor | Plasma and PBMC |
| 13 | Male | 57 | - | Healthy donor | Plasma and PBMC |
| 14 | Male | 47 | - | Healthy donor | Plasma and PBMC |
| 15 | Male | 64 | - | Healthy donor | Plasma and PBMC |
